# *prolfqua*: A Comprehensive *R*-Package for Proteomics Differential Expression Analysis

**DOI:** 10.1021/acs.jproteome.2c00441

**Published:** 2023-03-20

**Authors:** Witold E. Wolski, Paolo Nanni, Jonas Grossmann, Maria d’Errico, Ralph Schlapbach, Christian Panse

**Affiliations:** † Functional Genomics Center Zurich (FGCZ)−University of Zurich/ETH Zurich, Winterthurerstrasse 190, CH-8057 Zurich, Switzerland; ‡ Swiss Institute of Bioinformatics (SIB) Quartier Sorge−Batiment Amphipole, 1015 Lausanne, Switzerland

**Keywords:** proteomics, statistical software, differential expression analysis

## Abstract

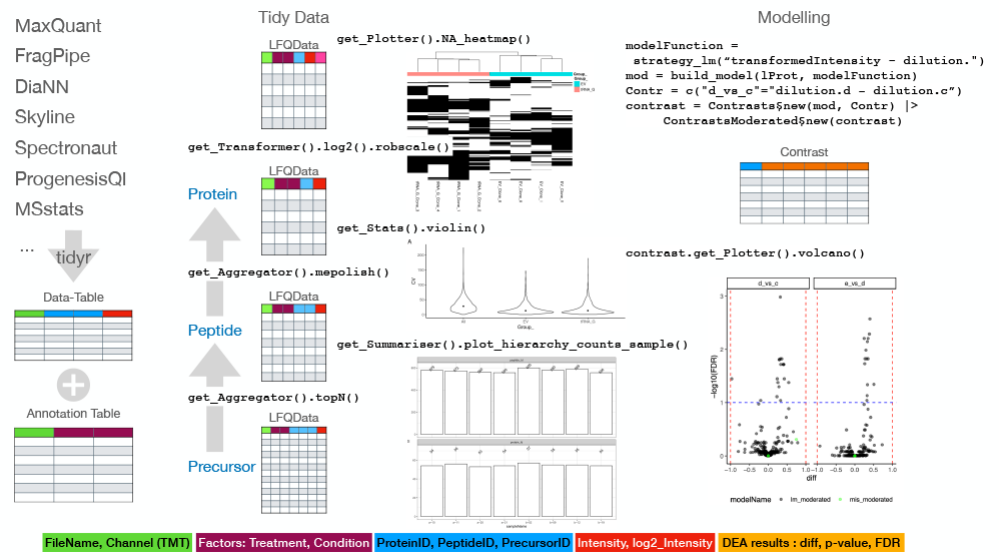

Mass spectrometry is widely used for quantitative proteomics studies, relative protein quantification, and differential expression analysis of proteins. There is a large variety of quantification software and analysis tools. Nevertheless, there is a need for a modular, easy-to-use application programming interface in *R* that transparently supports a variety of well principled statistical procedures to make applying them to proteomics data, comparing and understanding their differences easy. The *prolfqua* package integrates essential steps of the mass spectrometry-based differential expression analysis workflow: quality control, data normalization, protein aggregation, statistical modeling, hypothesis testing, and sample size estimation. The package makes integrating new data formats easy. It can be used to model simple experimental designs with a single explanatory variable and complex experiments with multiple factors and hypothesis testing. The implemented methods allow sensitive and specific differential expression analysis. Furthermore, the package implements benchmark functionality that can help to compare data acquisition, data preprocessing, or data modeling methods using a gold standard data set. The application programmer interface of *prolfqua* strives to be clear, predictable, discoverable, and consistent to make proteomics data analysis application development easy and exciting. Finally, the *prolfqua R*-package is available on GitHub https://github.com/fgcz/prolfqua, distributed under the MIT license. It runs on all platforms supported by the *R* free software environment for statistical computing and graphics.

## Introduction

Proteins carry out the most crucial functions and give structure to living cells. Hence, measuring changes in protein abundance is the subject of active research.^1^ Bottom-up mass spectrometric methods, where proteins are specifically and reproducibly digested into protein fragments—peptides, are employed to identify and quantify proteins in complex samples containing hundreds to thousands of proteins.^2,3^ The peptides are first separated by their chemical and physical properties using liquid chromatography (LC). Afterward, they are ionized, weighed, identified, and quantified using mass spectrometric techniques, e.g., electro-spray-ionization mass spectrometry (ESI-MS). Finally, peptide identification is achieved by fragmenting and matching the measured fragment masses to theoretical masses computed from known peptide sequences.^4−6^ For quantification, intact peptide ions^7,8^ or products of peptide ion fragmentation^9,10^ are counted and aggregated to obtain peptide abundances. Finally, the identified and quantified peptides are assigned to proteins based on protein sequence information.^11^

Proteomics quantification experiments enable monitoring of the relative abundances of thousands of proteins in biological samples. Most studies use parallel-group designs, where one or many treatment groups are compared to the control group.^12,13^ More recently, more complex experimental designs with an increasing number of samples have been studied, e.g., factorial designs and longitudinal studies (time series), sometimes with repeated measurements on the same subject.^14,15^ The data can be modeled using linear fixed-, mixed-, or random-effects models.^16^ Based on these models, tests can be applied to examine whether specific factors and factor interactions are significant; e.g., it can be tested if differences in protein abundance between groups are statistically significant.

An important aspect of mass spectrometric data are missing peptide and protein quantifications. Rubin^17^ classified missing data problems into three categories: missing completely at random (MCAR), missing at random (MAR), and missing not at random (MNAR). For instance, in data-dependent acquisition (DDA) MS, only a limited number of MS1 signals are selected for fragmentation and identified. Peptide quantification algorithms transfer identification information between MS1 features in different samples by aligning the data using retention time and mass information, thus reducing the amount of missing data. Nevertheless, highly abundant proteins can suppress the detection of other proteins in a sample, a MAR mechanism. Furthermore, a weak correlation between the number of missing measurements in a group and the abundance of the quantified protein can be observed, caused by the limit of detection (LOD), an MNAR mechanism.^18^

Several data analysis packages exist to model MS protein quantification experiments, e.g., *limma*,^19^*MSstats*,^20^*PECA*,^21^*msqrob2*,^22^ or *proDA*,^23^ to mention some, all implemented in *R*.^24^ Each of them has some unique features; for example, *MSstats* determines the statistical model from the structure of the sample annotation, which allows users with limited statistical knowledge to perform differential expression analysis (DEA). At the same time, *limma* enables the specification of a design matrix using a linear model formula and implements the empirical Bayes variance shrinkage method. The package *PECA* performs a roll-up of peptide level differences and peptide level *p*-value estimates obtained from *limma* or *PECA*, to protein level estimates. Furthermore, *msqrob2* combines robust linear models fitted to protein abundances and a quasibinomial generalized linear model fitted to peptide counts into Hurdle model. Finally, the *proDA* package implements a linear probabilistic dropout model to account for missing data without imputation.

Of note are the various approaches to handling missing observations, which are inherent in mass spectrometric bottom-up experiments. For instance, *MSstats* handles missing data by feature filtering and imputation. Other means of modeling missing observations are the Hurdle models discussed by Goeminne et al.,^25^ while the *R*-package *proDA* models missingness using probabilistic dropout models.^23^

We can use all the *R*-packages discussed when analyzing parallel-group designs using a single explanatory variable and contrasting groups. However, we can use only some of them to model factorial designs or repeated measurements. Table 1 gives an overview of the models and features these packages support. We see that, for instance, *limma* and *proDA* allow us to fit a comprehensive variety of models and test various hypotheses; however, good knowledge of the design matrix specification using the *R* formula interface is required.^26^

**Table 1:** Models supported^a^ by R-Packages used for differential protein expression analysis.

	pd	rm	eb	fd	int	mem	md
PECA	Y	Y	Y	NA	NA	NA	NA
limma	Y	Y	Y	Y	Y	NA	NA
MSstats	Y	Y	NA	Y	Y	Y	NA
proDA	Y	Y	Y	Y	Y	NA	Y
msqrob2	Y	Y	Y	Y	Y	Y	Y
prolfqua	Y	Y	Y	Y	Y	Y	Y

a pd, parallel design; rm, repeated measurements; fd, factorial design; int, interactions among factors; mem, mixed effect models; eb, empirical Bayes; md, missing data modelling (no imputation needed); Y, yes.

When developing the *R*-package *prolfqua,* we were inspired by the *R*-package *caret*(27) which enables us to call various machine learning (ML) methods and makes selecting the best ML algorithm for your problem easy. We aimed for a similar *R*-package for the DEA of quantitative proteomics data. However, the existing packages differ widely regarding supported designs, model specifications, and output formats. At the same time, they share the following features: fitting linear models to either peptide or protein abundances, determining differences among groups, and afterward applying empirical Bayes variance shrinkage. Therefore, the revised goal was to provide a modular object-oriented design, with *R* linear models as a core, and add features such as *p*-value aggregation, variance shrinkage, or modeling of missing observations.

Furthermore, *prolfqua* also includes methods specific to proteomics data. For example, we implemented strategies to estimate protein intensities from peptide intensities: top N,^28^ Tukey’s median polish,^29^ and robust linear models.^25^ Peptide or protein abundances can then be scaled and transformed using robust scaling, *quantile* normalization, or *vsn* to remove systematic differences among samples and heteroscedasticity. In this respect, *prolfqua* can be compared with *R*-packages such as *DEP*(30) or *POMA*(31), which support the entire DEA pipeline.

Since group sizes are relatively small, typically with four or five subjects per group, the high power of the tests is a relevant criterion to assess the modeling method. The quantified proteins can be ranked using the estimated fold-change, *t*-statistics, or scaled *p*-value and subjected to gene set enrichment (GSEA) or over-representation analysis^32^ to determine up or downregulated groups of proteins. Furthermore, the statistical model must provide an unbiased estimate of the false discovery rate (FDR) to manage expectations when selecting protein lists for follow-up experiments. We will use the partial area under the receiver operator curve (ROC) to assess the power of the tests and compare the FDR with the false discovery proportion (*FDP*). We use the *IonStar*(33) and *CPTAC*(34) data sets, processed with *MaxQuant* and *FragPipe*, to benchmark the modeling methods implemented in *prolfqua* and to compare our results with those of *MSstats*, *msqrob2*, and *proDA*. Although other benchmark data sets exist,^35,36^ the *IonStar* data set has the advantage that the expected differences, for the spike in proteins, among groups are small compared to other benchmark data sets, making DEA more difficult and enabling us to see performance differences among the modeling methods.

## Methods

### Implementation

**Figure 1:**
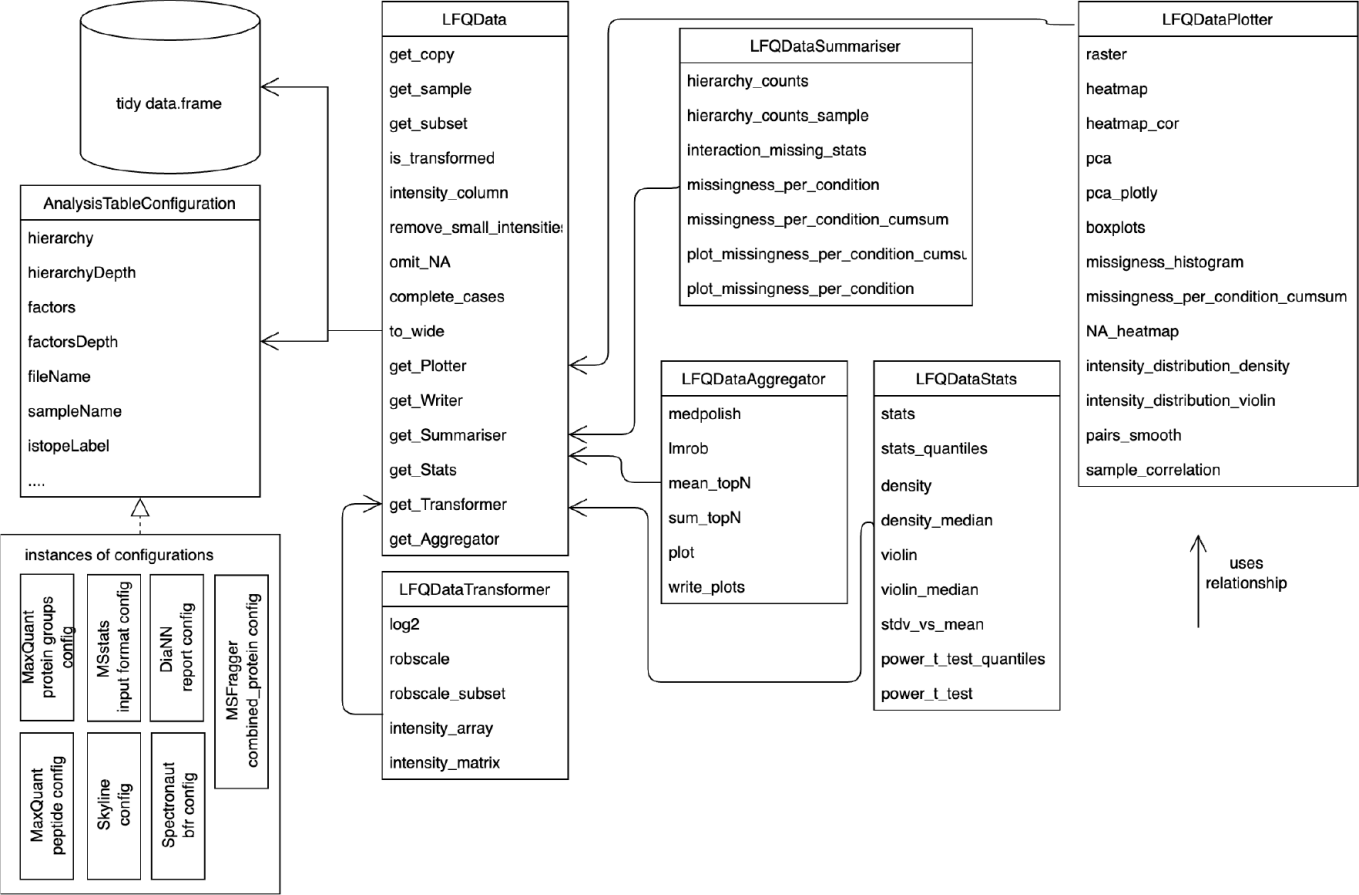
Class diagram of classes representing the proteomics data. The LFQData class encapsulates the quantitative proteomics data stored in a table of tidy data. An instance of the AnalysisTableConfiguration class specifies a mapping of table columns to sample names, peptide or protein identifiers, explanatory variables, and response variables. The LFQDataPlotter class and other classes decorate the LFQData class with additional functionality. For instance, the LFQDataStats and LFQDataSummary reference the LFQData class and group methods for variance and sample size estimation or summarizing peptide and protein counts. Furthermore, the LFQDataTransformer and LFQDataAggregator classes group functions for data normalization and estimating protein from peptide intensities.

**Figure 2:**
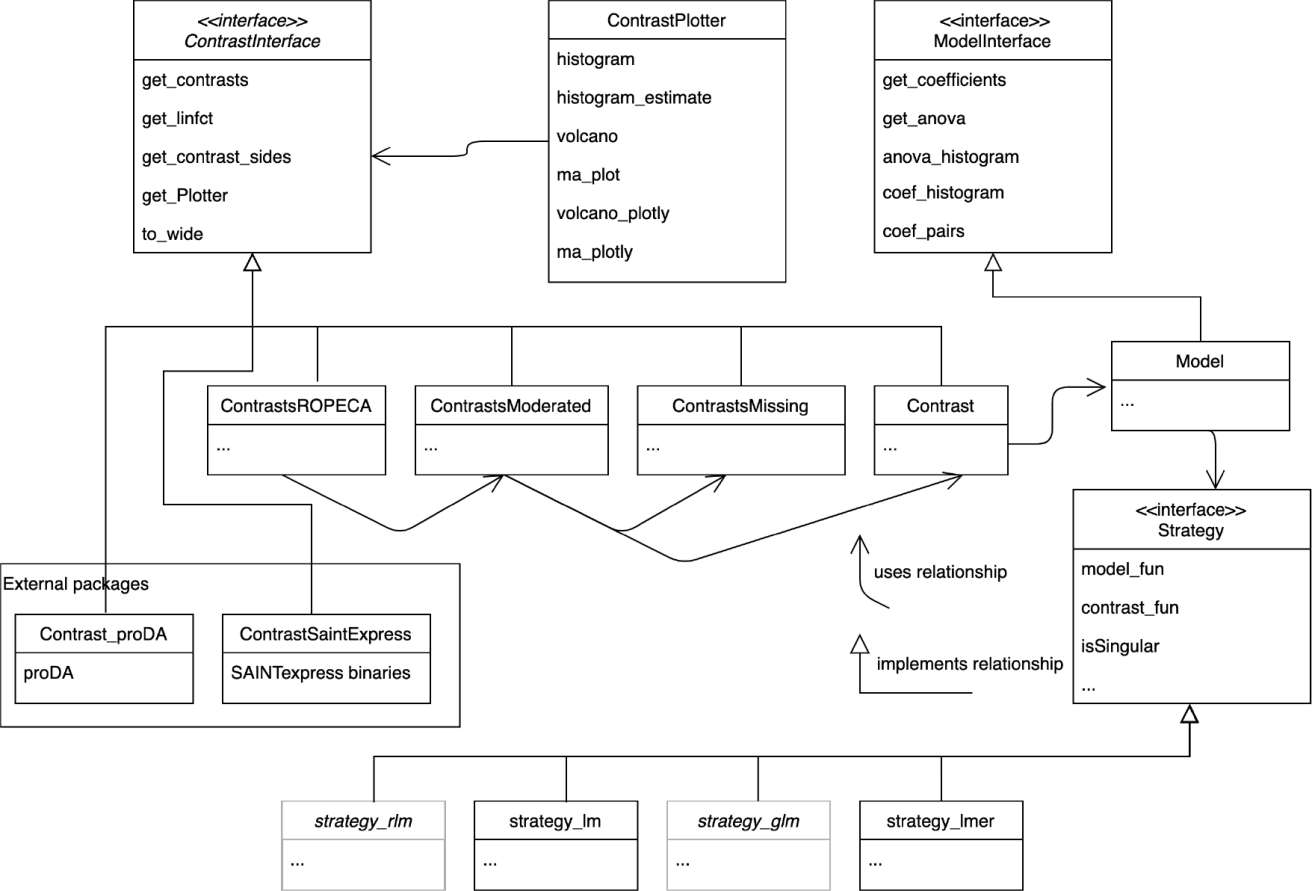
Unified modeling language (UML) diagram of modeling- and contrast-related classes. Different strategies, e.g., *lm*, *lmer*, and *glm* (Table 2), reference methods to fit models, and compute contrasts. The model builder method fits the statistical model given the data and a strategy. The models are used to analyze variance (ANOVA) or to estimate contrasts. All classes estimating contrasts implement the *ContrastsInterface*. Results of external tools, e.g., *SAINTexpress*, or *proDA* are adapted to implement the Contrasts interface.

We store all the data needed for analysis in a data frame as tidy data; i.e., every column is a variable, every row an observation, and every cell a single value.^37^ Using an R6^38^ configuration object (Figure 1), we specify which variable is in which column making it easy to integrate new inputs in *prolfqua* if provided as tidy data. For example, to visualize tidy Spectronaut,^39^ DiaNN,^10^ Skyline^40^ outputs, or data in *MSstats*(20) format, only a few lines of code are needed to update the *prolfqua*AnalysisTableConfiguration configuration. The configuration encapsulates the differences among the various input formats in column names and enables the using methods without additional arguments. An example code for creating a *FragPipe*(7) configuration can be found in Section S3, “Creating a Prolfqua Configuration”. We implemented methods that transform the data into tidy data for popular software like *MaxQuant*,^8^ or *FragPipe*, which stores the same variable, e.g., intensity, in multiple columns, one for each sample. Relying on the tidy data table enables us to interface with many data manipulation, visualization, and modeling methods, implemented in base *R*(24) and the tidyverse^41^ easily. We use R6 classes to structure the functionality of the package (see Figures 1 and 2). R6 classes are well supported by command-line completion features (see Figure S8 in the SI) in *RStudio*,^42^ and help to implement argument-free functions.

*R*’s formula interface for linear models is flexible, widely used, and well documented.^26,43^ We use the formula interface to specify the models, making it easy to reproduce an analysis performed with *prolfqua* in other statistical programming languages. In addition, we implement features specific to high-throughput experiments, such as the empirical Bayes variance and *p*-value moderation, which utilizes the parallel structure of the protein measurements and the analysis.^19^ We also compute probabilities of differential protein regulation based on peptide-level models.^21^ We used R6 classes to encapsulate the statistical modeling functionality in *prolfqua* (see Figure 2). We specify a contrast interface (ContrastsInterface). Several implementations allow the performance of DEA given linear or mixed effect models (Contrasts), variance shrinkage (ContrastsModerated), or to estimate contrasts in cases when observations are missing for an entire group of samples (ContrastsMissing). Further implementations of the interface encapsulate and integrate DEA results of external tools such as *proDA* or *SAINTexpress*(44) used to analyze data from protein interaction studies.

**Table 2:** prolfqua Functions That Can Be Used to Fit Various Models

prolfqua functions	model
strategy_lm, Contrasts	linear modeling of peptide or protein abundances and group difference estimation
strategy_lmer, Contrasts	mixed effect modeling of peptide or protein abundances and group differences estimation
ContrastsMissing	group difference estimation when no observations in one of the groups
ContrastsROPECA	estimating group differences for proteins by summarizing peptide differences
ContrastsModerated	empirical Bayes variance shrinkage for group difference estimates (limma)
runSaint, ContrastsSAINTexpress	protein interaction scoring (SAINTexpress)
strategy_proDA,^a^ ContrastsProDA	adapter to the probabilistic dropout model implemented in proDA

a In development.

### Data Sets for Benchmarking

#### IonStar

To evaluate the performance of DEA, we use the *IonStar* benchmark data set,^33^ available from the Proteomics Identifications Database (PRIDE) identifier PXD003881. *DH*5*α Escherichia coli* lysate was spiked in human pancreatic cancer cells (Panc-1) lysate at five levels: 3%, 4.5%, 6%, 7.5%, and 9% *E. coli*. We annotated these dilutions from smallest to largest with the letters *a*–*e*. By comparing the various dilutions, we obtain different effect sizes; e.g., when comparing dilution *e* (9%) against dilution *d* (7.5%), the expected difference is 1.2 for *E. coli* proteins and 1 for human proteins. There are four technical replicates for each dilution, hence 20 raw files in total. To compare the performance of the various methods implemented in *prolfqua*, we use only the contrasts resulting in minor differences Δ = (1.20, 1.25, 1.30, 1.50), because for bigger differences, all models perform similarly.

#### IonStar/MaxQuant

We processed the raw data of the *IonStar* data set using *MaxQuant*(8) Version 1.6.10.43, with *MaxQuant* default settings for Orbitrap data. The text files generated by *MaxQuant* are available in the *prolfquadata R*-package.^45^*MaxQuant* produces various output files which can be used for DEA. We are using the quantification results reported in the “peptide.txt” file for DEA. However, *MSstats* is using the “evidence.txt” file for the DEA.

#### IonStar/FragPipe

We processed the raw data of the *IonStar* data set using *FragPipe*(7) Version 14, with the default workflow for label-free quantification with match between runs enabled. The text files generated by *FragPipe* are available in the *prolfquadata R*-package.^45^ Similarly to *MaxQuant*, the *FragPipe* software produces various outputs which can be used for DEA. We used the total protein intensities reported in the “combined_protein.tsv” file as input for the DEA and called this data set IonStar/FragPipe/combined_protein.tsv. Alternatively, we benchmarked the DEA using the “MSstats.tsv” file as input and called this data set IonStar/FragPipe/MSstats.tsv.

#### CPTAC/MaxQuant

We used the CPTAC data set, available in the *R*-package *msdata*, and described in reference (22). In brief, the Sigma Universal Protein Standard mixture 1 (UPS1) containing 48 different human proteins was spiked in a protein background of 60 ng/μL *Saccharomyces cerevisiae* strain BY4741. Two different spike-in concentrations were used, 6A (0.25 fmol UPS1 proteins/μL) and 6B (0.74 fmol UPS1 proteins/μL). Three replicates are available for each concentration. The data were searched with *MaxQuant* version 1.5.2.8.

### Data Preprocessing for Model Comparison

The peptide abundances (from the *MaxQuant peptide.txt* file) were log_2_ transformed and subsequently scaled, where median and the mean absolute deviation was obtained from the human proteins only. We removed *one-hit wonders*, i.e., proteins with a single peptide assignment. Protein abundances are inferred from the peptide intensities using Tukey’s median polish. Finally, we fitted the fixed effect linear models, the dropout model *proDA* to protein abundances, the mixed effect linear model, the *ROPECA* model, and the hurdle model implemented in *msqrob2* to peptide intensities.

### Benchmark Metrics

The *IonStar* data set contains *Homo sapiens* proteins with constant concentrations and *E. coli* proteins with varying concentrations. We know that for *H. sapiens* proteins, the difference β between two dilutions should be β = 0, while for *E. coli* proteins, we know that the difference between dilutions should be β ≠ 0.

We can use various statistics to examine the alternative hypothesis β ≠ 0: the contrast estimate, i.e., the log_2_ fold-change β, the *t*-statistic , or the *p*-value and moderated *p*-value. For each statistic and each value of the statistics we then compute a confusion matrix (see Table 3). From the confusion matrix we obtain measures such as true positive rate (*TPR*), false positive rate (*FPR*), or false discovery proportion (*FDP*) which are given in Table 3 with
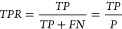
1
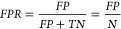
2
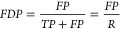
3

**Table 3:** Confusion Matrix^a^

prediction/truth	*E. coli*	*H. sapiens*	total
beta != 0	TP	FP	R
beta == 0	FN	TN	
total	P	N	m

a TP, true positive; FP, false positive; FN, false negative; TN, true negatives; P, all positive cases (all *E*. *coli* proteins); N, all negative cases (all *H*. *sapiens* proteins); m, all proteins.

In order to compute the confusion matrices based on the *p*-value we first need to rescale it (see eq 11).

By plotting the *TPR* versus the *FPR* we obtain the receiver operator characteristic curve (ROC curve).^46^ The area under the curve (*AUC*) or partial areas under the curve (*pAUC*), at various values of the *FPR*, are measures of performance derived from the ROC curve. Using these measures, we can compare the performances of the statistics for a single model or the various models and test if the differences are statistically significant, using a test to compare ROC curves.

### Modeling

#### Robust Scaling of the Data

Välikangas et al.^47^ discuss and benchmark various methods of peptide or protein intensity normalization, such as variance stabilizing normalization^48^ or quantile normalization.^49^ In this work, we use a robust version of the *z*-score, where instead of the mean we use the median *x̃*, and instead of the standard deviation we use the median absolute deviation *S̃*:
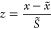
4

Because we need to estimate the differences among groups on the original scale, we must multiply the *z*-score by the average standard deviation of all the *n* samples in the experiment.
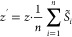
5

To apply this transformation, we need to estimate two parameters per sample; therefore, it works for experiments with thousands of proteins and experiments where only a few hundred proteins per sample are measured. For the Ionstar data set, we used the intensities of *H. sapiens* proteins, whose concentrations do not change, to determine *x̃* and *S̃* and then applied it to all the intensities (including *E. coli*) in the sample.

#### Estimating Differences between Groups

Given a linear model *y* = *βX*, we can compute the difference β_*c*_ between two groups by the dot product of weights *c* and model parameters β, where *c* is a column vector with as many elements as there are coefficients β in the linear model. If *c* has 0 for one or more of its rows, then the corresponding coefficient in β is not involved in determining the contrast.^50^

The difference estimate β_*c*_ is given by the dot product
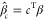
6and the variance of β_*c*_ by
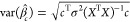
7with *X* being the design matrix. The degrees of freedom for the contrast are equal to the residual degrees of freedom of the linear model. For estimating contrasts from mixed effects models we used the function contest implemented in the *R*-package *lmerTest* and used the Satterthwaite^51^ method to estimate the denominator degrees of freedom. These methods are available in the class Contrast (see Figure 2).

The package *prolfqua* provides functions to determine the vector of *parameter* weights *c*, from a linear model and a contrast specification string. In section Material S10 in the SI, we provided an example of how to specify contrasts for a data set with two explanatory variables and an interaction term.

#### Contrast Estimation in the Presence of Missing Data Using LOD

Missing observations lead to different group sizes, which results in unbalanced designs. Linear and mixed effect models can handle unbalanced designs. As long as at least one observation in a group is available, and sufficient observations to estimate the variance are available, they will produce unbiased estimates. Therefore, no imputation is needed.

However, if there is no observation in a group the model fit fails. For example, suppose a protein is unobserved in all the samples of a group. In that case, a plausible explanation is that the protein abundance is below the limit of detection (LOD) of the MS instrument. In such a case, we will substitute the group mean using the expected protein abundance *A* at the LOD *A*_*LOD*_. To estimate *A*_*LOD*_ we are using the protein abundances of those groups where the protein was observed in only a single sample (see section Material S8 “Estimating *A*_*LOD*_”, in the SI). Typically there are many such cases, and hence we take the median.

When computing differences Δ among two groups *a* and *b*, we will use either the group mean *a̅* or *b̅* estimated from the data. However, if for instance no observations are present in group *b*, we will use . Furthermore, if , we also set , or more formally
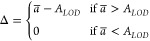
We use the pooled variance in all groups to estimate the protein variance, assuming they are the same. The pooled variance *s*_*p*_^2^ is given by
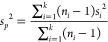
8with *n*_*i*_ the number of observations, and *s*_*i*_ the standard deviation in each group. The standard deviation for the *t*-statistics is then given by
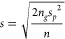
9

Where *n*_*g*_ is the number of groups, and *n* is the number of observations. If variance cannot be estimated for a protein, because there are too few observations in other groups, we use the median pooled variance of all other proteins in the data set. This method is implemented in the class ContrastsMissing (see Figure 2).

#### *p*-Value Moderation

From the linear and the mixed effect models, we can obtain the residual standard deviation σ, and degrees of freedom *df*. Smyth^52^ discuss how to use the σ and *df* of all models to estimate the corresponding priors and posterior σ̃. These can be used to moderate the *t*-statistics by
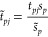
10

We implemented this method in the class ContrastModerated (Figure 2).

#### Summarizing Peptide Level Differences and *p*-Values on Protein Level

To summarize peptide level models to protein models, we apply the method suggested by Suomi and Elo^21^ that uses the median scaled *p*-value of the peptide models and the cumulative distribution function of the Beta distribution (CDF) to determine a regulation probability of the protein.

To obtain the *p̃* of a protein we first rescaled the peptide *p*-values by taking the sign of the fold-change β̂ into account, i.e.:
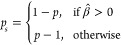
11

Afterward, the median scaled *p*-value  is determined and, using the transformation below, transformed back onto the original scale:
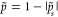
12

Because we use the median with the *i*th order statistic , we parametrize the CDF of the Beta distribution with  and . We implemented this method in the class ContrastROPECA (Figure 2).

## Results and Discussion

### Example Analysis Workflow

**Figure 3:**
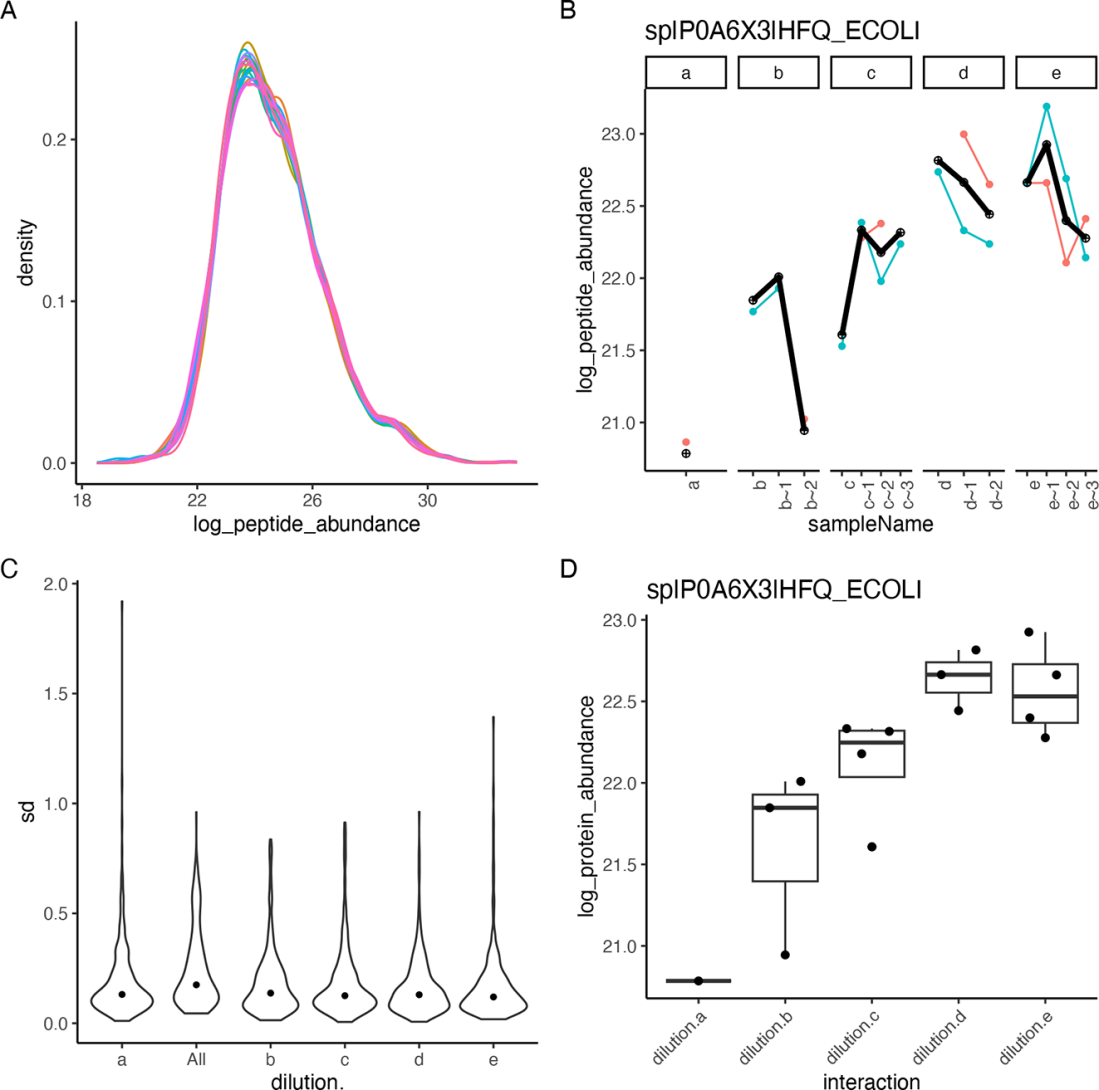
(A) Density plot of peptide intensity distributions for 20 samples. For each sample a line with a different color is shown. (B) Peptide intensities for protein HFQ_ECOLI are shown using lines of different colors, and the protein intensity estimate is shown using a fat black line. (C) Distribution of standard deviations of all proteins in each dilution group (a–e) and overall (all). (D) Distribution of protein intensities of Protein HFQ_ECOLI in each dilution group.

The code snippets in this section demonstrate how a DEA workflow can be implemented using the *prolfqua R*-package (see Material S1 "How to Install *prolfqua* and *prolfquabenchmark*" in the SI). To speed up the computation of these examples, we use a subset of the Ionstar data set generated by randomly selecting 400 proteins. First, we remove all proteins with a single peptide and all observations for which *MaxQuant* reports zero intensities, leaving 332 proteins. Next, peptide abundances are log_2_ transformed and robust *z*-score scaled using the method robscale. Then, using the LFQDataPlotter class, we show the distribution of the normalized peptide abundances in Figure 3A. Afterward, protein intensities are estimated from peptide intensities using Tukey’s median polish. Figure 3B shows the peptide intensities and the estimated protein intensities. Next, we compute the standard deviation of all the proteins in each group and display their distribution using violin plots (Figure 3C). Finally, we create a box plot (Figure 3D) showing the abundance of one protein.
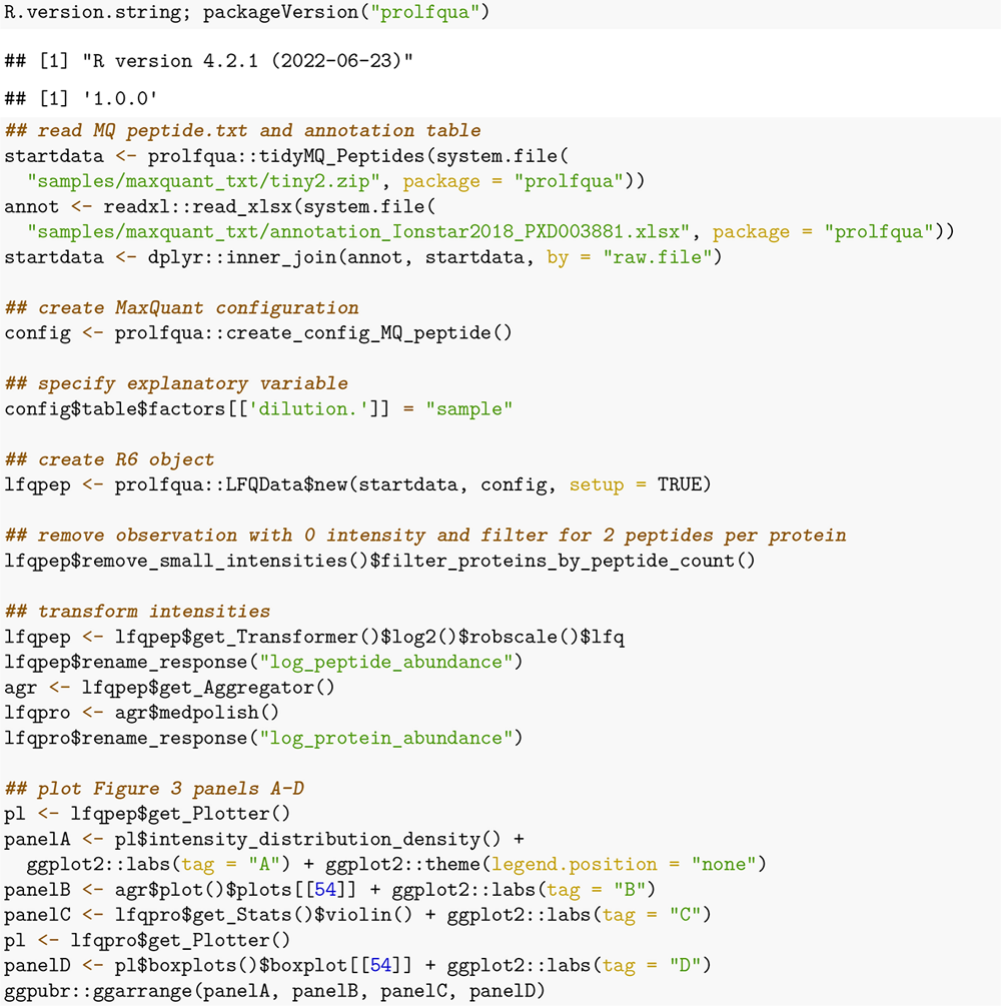


**Figure 4:**
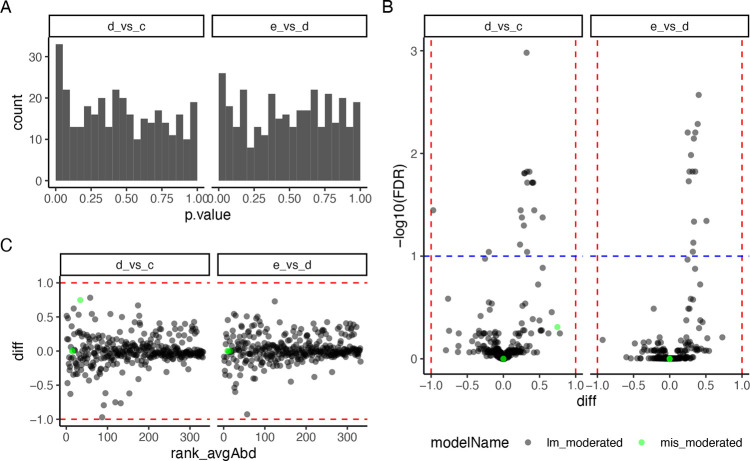
(A) Histogram showing the distribution of *p*-values for 332 proteins for contrasts “e_vs_d” and “d_vs_c”. (B) Volcano plot showing −log_10_ transformed *FDR* as a function of the difference between groups for 332 proteins. With black dots, we show effect size and FDR estimates obtained from the linear model, while in green, we plot those obtained using imputation. (C) Difference between groups, as a function of the rank of the abundance of the proteins.

The following code example illustrates how we compute differences among groups. First, the linear model and the differences are specified. Afterward, the model is fitted to the data using the build_model function. Next, we estimate the contrasts from the linear model using the Contrasts class or directly from the data using the ContrastsMissing class. Afterward, we apply *t*-statistic moderation using the ContrastModerated class. Finally, the merge_contrasts_results function merges both sets of contrast estimates, preferring the one obtained from the linear model if both are available. Then we create the plots shown in Figure 4. Figure 4A shows the distribution of the *p*-values, Figure 4B is the volcano plot for each comparison, and Figure 4C is a Bland–Altman plot reporting the difference between groups as a function of the rank of the protein abundance.
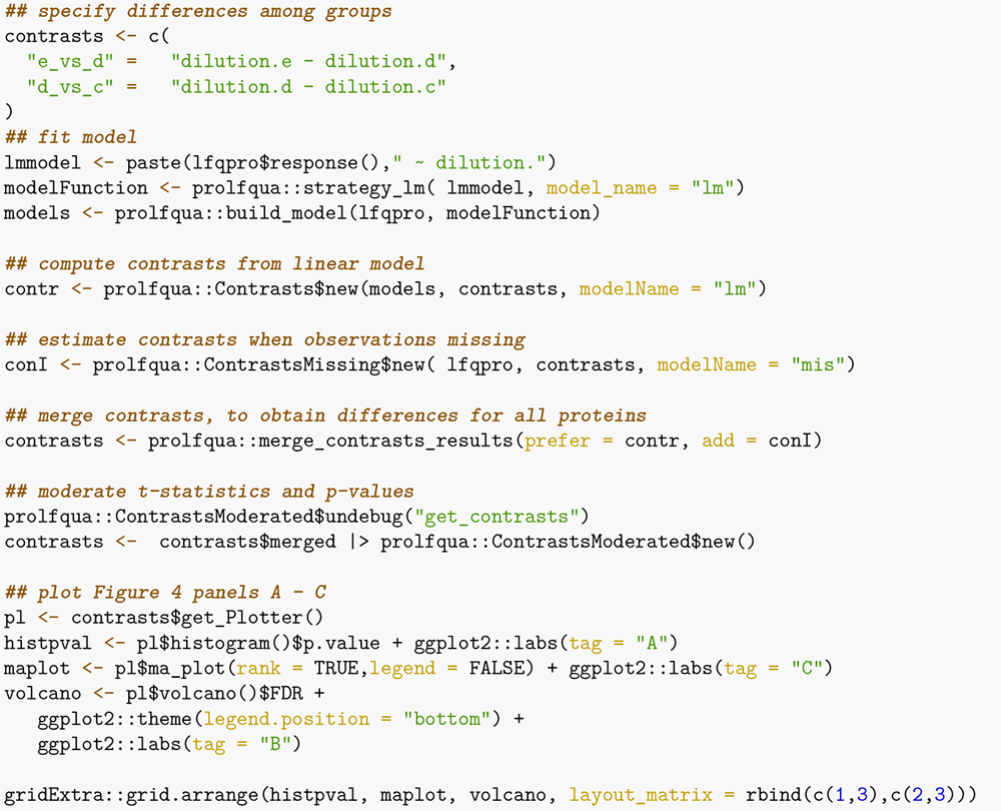


The *R* linear and mixed effect models allow modeling parallel designs, repeated measurements, factorial designs, and many more features. Models in *prolfqua* are specified using *R*’s linear and mixed model formula interface. Therefore, knowledge of the *R* regression model infrastructure^43,53^ is advantageous when using our package. Furthermore, this glass box approach should make it easy to reimplement an analysis performed with *prolfqua* using base *R* or other programming languages by reading the analysis script. However, in the package documentation, we showcase how a user, without this knowledge, can analyze experiments with a parallel-group design and a factorial design.

Using the data frame of tidy data ensures interoperability with other proteomics-related packages that manage their data with tidy-tables, e.g., *protti*.^54^ To simplify the integration of *prolfqua* with Bioconductor-based workflows, we provide a method that converts the LFQData class into a *SummarizedExperiment*. The use of R6 classes, which encapsulate the configuration and the data, allow for writing very concise code where functions can have few arguments. Autocompletion support for R6 classes in the *RStudio* editor makes it easy for novices to explore *prolfqua*’s functionality (see Figure S8 in the SI).

To ease the usage barriers of the *R*-package to users not familiar with statistics and *R* programming, we developed an application based on the *prolfqua* package into our data management platform B-Fabric.^55,56^ The B-Fabric system runs a computing infrastructure controlled by a local resource management system that supports cloud-bursting.^57^ This integration enables users to select the input data and basic settings in a graphical user interface (GUI). This way, *prolfqua,* and B-Fabric help scientists meet requirements from funding agencies, journals, and academic institutions while publishing their data according to the FAIR^58^ data principles. We are working on creating a shiny standalone application with the described functionality and making it available soon.

### Benchmarking Modeling Approaches

Using a benchmark data set with known ground truth (see the Methods section), we assessed the performance of different modeling approaches implemented in *prolfqua*, *MSstats*, *proDA*, and *msqrob2*. Table 4 summarizes which methods we have evaluated, which *MaxQuant* files we used as input, and if the models are fitted to peptide or protein intensities. We make the *R*-markdown files to replicate the benchmarking available at *prolfquabenchmark* (see Material S2 “Benchmark Vignettes (IonStar/MaxQuant)” in the SI).

**Table 4:** All Benchmarked Models^a^

label	description	abundance	input file
MSstats	preprocess with default parameters	precusor	evidence.txt
msqrob2	merge of msqrobHurdleIntensity and msqrobHurdleCount (msqrobHurdle)	protein and peptide	peptide.txt
proDA	probabilistic dropout model	protein	peptide.txt
prolfqua_missing	ContrastsMissing, ContrastsModerated	protein	peptide.txt
prolfqua_lm_mod	strategy_lm, Contrasts, ContrastsModerated	protein	peptide.txt
prolfqua_merged	addContrastResults(prefer = proflqua_lm_mod, add = prolfqua_missing)^b^	protein	peptide.txt
prolfqua_mix_eff_mod	strategy_lmer, Contrasts, ContrastsModerated	peptide	peptide.txt
prolfqua_ropeca	strategy_lm, Contrasts, ContrastsModerated, ContrastsROPECA	peptide	peptide.txt

a Label, name of the method; description, functions used in the respective packages; abudances, indicates if model is fitted to peptide or protein abundances; input file, name of MaxQuant file used as input.

b “prolfqua_merged”, augments estimates which are missing in “prolfqua_lm_mod” with those from “prolfqua_missing”.

The IonStar/MaxQuant data set (see the Methods section) captures only the variance from the chromatography, electrospray, and mass spectrometric measurements since only technical replicates are available for each dilution. Therefore, essential sources of variation typically present in other experiments, such as biochemical and biological ones, are not measured. Furthermore, this data set with a parallel-group design does not allow for benchmarking models with interactions. Thus, while we can extrapolate some of the results to more realistic data sets, we must be careful not to overinterpret our findings. Specifically, the observed variances will be higher in data sets with biological replicates, and the power will be lower for the same number of samples. Furthermore, the proportion of missing observations in real-life data sets might be higher or distributed differently in groups.

**Figure 5:**
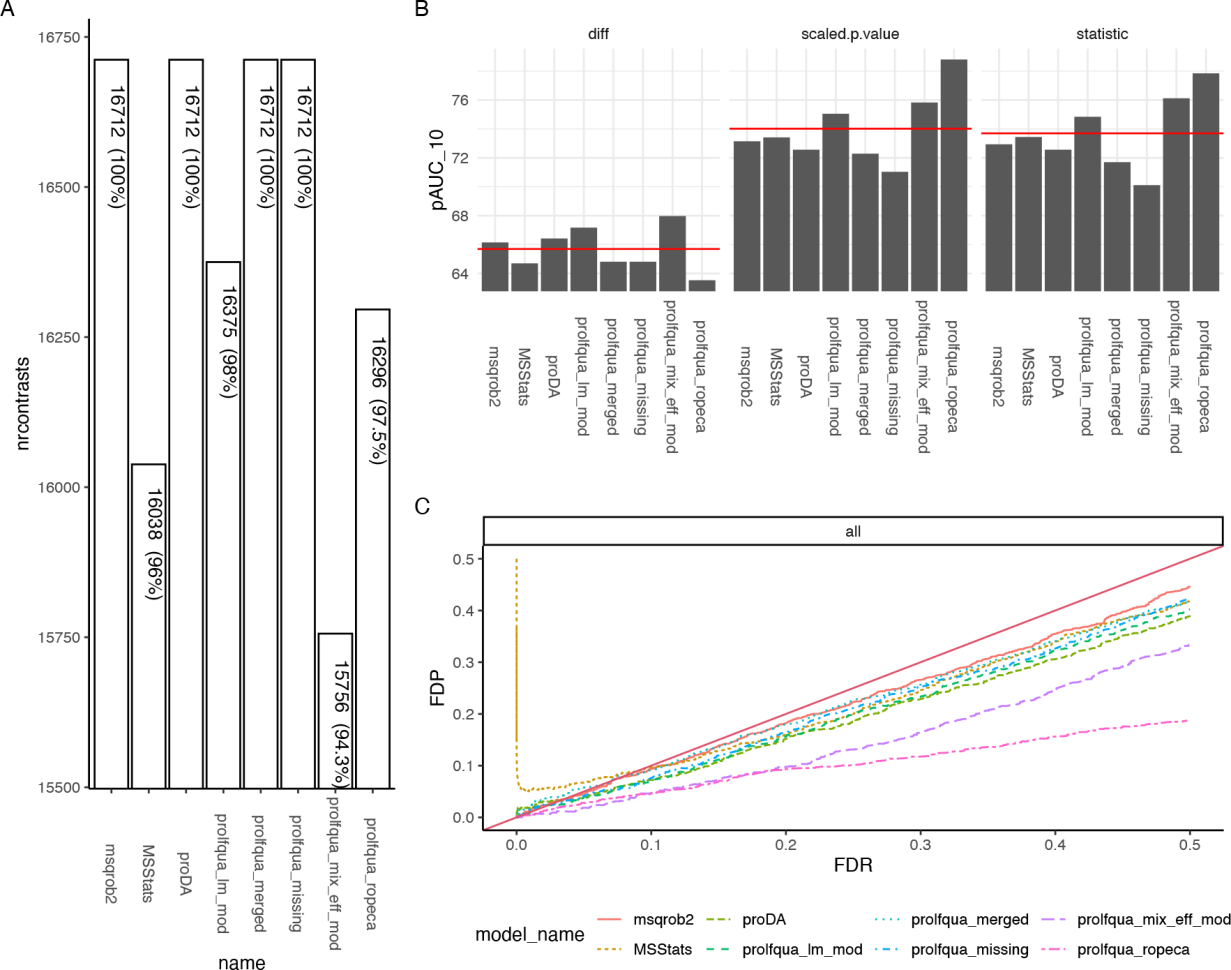
(A) Number of estimated contrasts for each modeling method (higher is better). (B) Partial area under the ROC curve at 10% FPR (*pAUC*_10_) for all contrasts and three different statistics: the difference among groups (diff, panel B left), the scaled *p*-value (sign(diff)·p.value) (scaled.p.value, panel B center), and the *t*-statistics (statistic, panel B right), where a higher *pAUC*_10_ is better. The red line indicates the average area under the curve of all methods. (C) Plot of the false discovery proportion (*FDP*) as a function of the *FDR*. Ideally, the *FDR* should be equal to the *FDP*. Therefore, larger distances from the diagonal are worse.

When comparing DEA performance, a relevant parameter is the number of differences among conditions a method can estimate (see Figure 5A). For each protein, we tried to determine four differences [Δ = (1.20, 1.25, 1.30, 1.50)], and therefore, given 4178 proteins with at least two peptides, there are, in total, 16712 possible differences. Since *msqrob2*, *proDA*, *proflqua_missing*, and *prolfqua_merged* directly model missing observations, they estimate all possible contrasts. However, some models fail to estimate differences when abundances are unobserved or rely on imputation. For instance, when using the mixed effect models, sensitive to missing data, we estimate the fewest number of contrasts with 15756.

The benchmark functionality of *prolfqua* includes receiver operator curves (ROC) and computes partial areas under those curves (*pAUC*) and other scores, e.g., the false discovery proportion *FDP*. Since the set of effect size estimates will differ for some methods, e.g., 16712 vs. 15756 (see Figure 5A), this introduces a bias when computing receiver operator curves and the *pAUC*. Hence, to conclude that one method performs better, it does not suffice if the *pAUC* is greater, but the number of proteins with differential expression results needs to be equal or larger. However, for *proDA*, *msqrob2*, and *prolfqua_merged*, we can compare the *pAUC* to asses which method performs best.

Figure 5B shows how various estimates obtained from the models, i.e., the difference between groups, *t*-statistics, and scaled *p*-values, allow identifying true positives (TP) given a false positive rate (FPR) of 10% by displaying the partial area under the ROC (*pAUC*_10_). Ordering proteins by the *t*-statistic or *p*-value leads to a higher *pAUC*_10_ than when ordering by the estimated difference among groups.

We can conclude that if we want to sort the proteins according to the likelihood of being differentially regulated to perform gene set enrichment analysis,^32^ the *t*-statistic is better suited than the fold-change estimate. When computing the *p*-values from the *t*-statistics, we incorporate the degrees of freedom, improving the inferences (see Figure 5B, center versus left). There is no such improvement for the mixed effect model. The reason is an erroneous denominator degree of freedom estimation for many proteins, a known problem in the case of mixed effect models. Furthermore, for the fixed effect linear model, the empirical Bayes variance shrinkage, as suggested by Smyth,^52^ consistently improves the ranking of proteins compared with the unmoderated estimates (not shown). However, since also for this method, a correct degree of freedom estimate is required, it does not work for mixed effect models.

Suppose an accurate estimate of the difference among groups is essential. In that case, among the models fitted to protein intensities, calculated using Tukey’s median polish, the *proDA* model performed best (see Figure 5B left). The dropout model more accurately models the posterior protein intensities, compared with *prolfqua_missing*, which uses a point estimate of the *LOD*. Furthermore, the *prolfqua_ropeca* model that first fits peptide level models and then summarizes differences performed worst. We speculate that the peptide-level outliers do not affect the protein estimates when using Tukey’s median polish method.

We also benchmark if the *FDR* obtained from a model is an unbiased estimate of the false discovery proportion *FDP*. Figure 5C shows the *FDP*, obtained from the confusion matrix, as a function of the *FDR* determined from the model. Most lines are below the diagonal, which indicates that the *FDR* estimates are modestly conservative for this particular benchmark data set. In the case of *MSstats*, we observe a high proportion of false discoveries for small *FDR* values. In the case of the *prolfqua_ropeca* method, the *FDR* estimates, obtained by applying the Benjamini–Hochberg correction to the Beta distribution-based regulation probabilities, strongly overestimate the *FDP*.

However, computing the *t*-statistics at the peptide level and then summarizing it for each protein using their median produces the highest *pAUC*_10_ scores among all the tested models (see Figure 5B *prolfqua_ropeca*). Furthermore, by using the Beta distribution to model the number of peptides observed, we can further improve the *pAUC* scores (see Figure 5B center). However, the properties of Beta-based probabilities need to be better understood; their distribution is not uniform under the null hypothesis (see section Material S9 “The probabilities produced by ROPECA are not *p*-values” in the SI). Therefore, the resulting *FDR* estimates are biased (see Figure 5C). Consequently, we cannot recommend this method if an unbiased estimate of *FDR* is essential, which is frequently the case. In addition, peptides are more strongly affected by missing values, reducing the number of contrasts we could estimate for the data set using this method (see Figure 5C).

The *R*-packages *proDA*, *msqrob2*, and *prolfqua* do not impute missing data but integrate them into the statistical model, while *MSstats* filters and imputes the data using an accelerated failure model. Despite imputation, *MSstats* estimates fewer group differences (16038) and does not achieve a higher *pAUC*_10_ (see Figure 5). Furthermore, Figure 5C shows that when using *MSstats*, the proportion of false discoveries might be very high for a low *FDR* because of false positives. Hence, augmenting the linear model for handle missing observations using the quasi-binomial generalized linear model, the dropout model, or estimating missing differences using the LOD simplifies the analysis pipeline since no imputation is needed and improves the quality of the estimates.

Of note, *MSStats* uses the *evidence.txt* file, while all the other methods use *peptide.txt* files as input (see Table 4). Furthermore, *MSstats* uses equalized medians normalization, while all the other methods use robust scaling (see the Methods section). These are possible confounding factors to consider. Finally, while *prolfqua*, as well as *proDA*, is highly modular, and to a lesser extent *msqrob2*, enabling us to use the same data preprocessing and normalization, *MSstats* is monolithic, making it unfeasible to use a preprocessing or normalization method not available in *MSstats*.

We obtained difference and FDR estimates for all proteins and comparisons, as shown in Figure 5A when using (a) the probabilistic dropout model (*proDA*), (b) the hurdle model (*msqrobHurdle*), and (c) *prolfqua_merged*. We observe that the performance of the scaled *p*-values or the *t*-statistics are comparable among these three methods (Figure 5B). We tested if there was a significant difference between the *pAUC*_10_ for all three methods, but did not reject the null hypothesis that there is no such difference (section Material S3 “DEA benchmark: IonStar/MaxQuant/peptide.txt - Significance test” in the SI). Also, the FDR estimates (Figure 5C) are comparable for all three methods.

Furthermore, all three models perform similarly when examined using a different benchmark data set CPTAC/MaxQuant (see the Methods section). For this data set, *proDA* performed slightly but not significantly better than *prolfqua* and *msqrobHurdle* (and section Material S4 “DEA benchmark: CPTAC/MaxQuant/peptide.txt” in the SI).

In addition, we examined the DEA performance when using protein intensities reported by quantification software *FragPipe* for the *IonStar* data set as input. Using protein abundances as input significantly simplifies the analysis and interpretation and might benefit from optimization implemented in the quantification software. However, we can only fit the *proDA* and *prolfqua_merged* (see Table 4) models to protein abundances, while *MSstats* and *msqrobHurdle* require peptide spectrum match or peptide level abundances. In this DEA benchmark, *prolfqua* performed slightly but not significantly better than *proDA* (section Material S5 “DEA benchmark: IonStar/FragPipeV14/combined_protein.tsv” in the SI).

Finally, we also compared the DEA performances when starting the analysis from the precursor abundances reported in the “MSstats.tsv” file, generated by *FragPipe* v14, from the *IonStar* data set. Since *MSstats*, *msqrob2*, *proDA*, and *prolfqua* all read MStstats.tsv files, we could eliminate a confounding factor, i.e., different input abundances (section Material S6 “DEA benchmark: IonStar/FragPipeV14/MSstats.tsv” in the SI). In this DEA benchmark, *msqrob2* and *prolfqua_merged* perform best but not significantly better than *proDA* or *MSstats*. Furthermore, by processing the *IonStar* data set with both *MaxQuant* and *FragPipe*, we can compare their performances (section Material S7 “Comparing DEA results for *MaxQuant* and *FragPipe*” in the SI).

We focused our benchmark on comparing the statistical modeling methods while we fixed the preprocessing steps. However, some of these steps are of utmost significance when performing differential expression analysis.^59^ One of them is the normalization of the abundances within the samples to remove systematic differences.^60^ The method used to infer proteins from peptide identifications^11^ and protein abundances from peptide abundances is an additional important factor.^28^ For instance, the original *proDA* publication uses MaxLFQ^61^ protein estimates. However, when using MaxLFQ abundances reported by *MaxQuant*, the *pAUC*_10_ is lower [*pAUC*_10_(*t*-statistics) = 66%] compared with results obtained when protein abundances are estimated from peptide abundances using Tukey’s median polish [*pAUC*_10_(*t*-statistics) = 72%]. Last but not least, the software^7,8^ used to identify and quantify proteins significantly contributes to the entire pipeline’s performance altering the number of identified proteins and the sensitivity and specificity of the differential expression analysis. In section Material S7 “Comparing DEA results for *MaxQuant* and *FragPipe*” in the SI, we compare DEA benchmarking results for the quantification software. While the number of proteins identified with two peptides is practically the same, the DEA benchmark performance differs significantly by ∼10% for the *pAUC*_10_ score. This difference is more significant than the differences due to the choice of the modeling method.

## Conclusion

*prolfqua* is a feature-rich, object-oriented, and modular *R*-package to analyze quantitative mass spectrometric data with simple or complex experimental designs. While other *R*-packages for differential expression analysis of proteins typically only implement one modeling approach, *prolfqua* supports various models (see Figure 2 and Table 2). Furthermore, the contrast specification is explicit and consistent for all models and allows for testing interactions. The modular design of *prolfqua* enables adding new features, e.g., generalized linear models to model the presence or absence of a protein quantification, or robust linear models, in the future. Furthermore, the developed framework can integrate other modeling methods, e.g., the probabilistic dropout model^23^ or accurate variance estimation.^62^ Hence, *prolfqua* enables the implementation of applications where the user can select an alternative normalization method, protein abundance estimation method, or DEA algorithms. Furthermore, this *R*-package can analyze other types of quantitative proteomics data, e.g., label-free DIA or labeling-based TMT data.

When comparing statistical modeling methods for the DEA, we assessed performance measures such as the number of estimated contrasts, the *pAUC*, and if the *FDR* is an unbiased estimate of the *FDP*. It is relevant that an analysis pipeline shows good performance in all these categories. The examined models *prolfqua_merged*, *proDa*, and *msqrob2* performed well in all these categories. Leveraging these computational experiments, we can provide the following advice: (i) Estimate protein abundances from peptide abundances using a robust or nonparametric regression method. (ii) Fit linear models to protein abundances. (iii) Do not impute missing observation but statistically model missingness to estimate parameters, i.e., group differences. (iv) Explicitly report the model used. (v) If the measurements are correlated, as for technical replicates, mixed effect models might work if the sample sizes are large; if not, aggregate the replicates and fit a linear model instead. (vi) If you use fixed effect linear models, apply variance moderation to improve the *t*-statistics and *p*-value estimates. (vii) If you want to sort your protein lists to perform gene set enrichment analysis, use the *t*-statistic instead of the difference.

## Supporting Information Available

Material S1: How to Install *prolfqua* and *prolfquabenchmark*; Material S2: Benchmark Vignettes; Material S3: DEA benchmark IonStar/MaxQuant/peptide.txt -S ignificance test; Material S4: DEA benchmark : CPTAC/MaxQuant/peptide.txt; Material S5: DEA benchmark : IonStar/FragPipeV14/combined_protein.tsv; Material S6: DEA benchmark : IonStar/FragPipeV14/MSstats.tsv; Material S7: Comparing DEA results for *MaxQuant* and *FragPipe*; Material S8: Estimating *A*_*LOD*_; Material S9: Probabilities produced by ROPECA, which are not *p*-values; Material S10: Specifying Contrasts for Models with two Factors and Interaction Term; and Material S11: Creating a prolfqua configuration (PDF)

## Abbreviations

API: application programming interface
AUC: area under the curve
CDF: cumulative distribution function
DEA: differential expression analysis
DIA: data independent acquisition
ESI-MS: electro-spray-ionization mass spectrometry
FAIR: findable, accessible, interoperable, and reusable
*FDP*: false discovery proportion
*FDR*: false discovery rate
*FP*: false positive
*FPR*: false positive rate
LC: liquid chromatography
LC-MS: liquid chromatography followed by mass spectrometry
LOD: limit of detection
MAR: missing at random
MCAR: missing completely at random
ML: machine learning
MS: mass spectrometry
pAUC: partial area under the curve
*pAUC*_10_: partial area under the curve for an FPR of 0–10%
ROC: receiver operator curve
TP: true positive
TMT: tandem mass tag
UML: unified modeling language
