# Supplementary material for: prolfqua: A Comprehensive R-Package for Proteomics Differential Expression Analysis
Source: J Proteome Res. 2023 Mar 20;22(4):1092–104. doi: 10.1021/acs.jproteome.2c00441 (PMC10088014; doi:10.1021/acs.jproteome.2c00441)
Supplement: Supplementary file 1 — pr2c00441_si_001.pdf [file pr2c00441_si_001.pdf]

Supporting Information
prolfqua: A Comprehensive R-package for Proteomics Differential Expression Analysis

Witold E. Wolski[1,2,*] Paolo Nanni[1] Jonas Grossmann[1,2] Maria d'Errico[1,2]
Ralph Schlapbach[1] Christian Panse[1,2]

Abstract

This document contains the supplements for the prolfqua manuscript available through https://doi.org/10.1101/2022.06.07.494524.

Contents

Material S1: How to Install *prolfqua* and *prolfquabenchmark* **S–2**

Material S2: Benchmark Vignettes (IonStar/MaxQuant) **S–2**

Material S3: DEA benchmark IonStar/MaxQuant/peptide.txt - Significance test **S–2**

Material S4: DEA benchmark : CPTAC/MaxQuant/peptide.txt **S–2**

Material S5: DEA benchmark : IonStar/FragPipeV14/combined_protein.tsv **S–5**

Material S6: DEA benchmark : IonStar/FragPipeV14/MSstats.tsv **S–7**

Material S7: Comparing DEA results for *MaxQuant* and *FragPipe* **S–7**

Material S8: Estimating A_{LOD} **S–10**

Material S9: The probabilities produced by ROPECA are not p-values **S–10**

Material S10: Specifying Contrasts for Models with two Factors and Interaction Term **S–11**

Material S11: Creating a prolfqua configuration **S–12**

Miscellaneous **S–13**

Session info **S–16**

[1] Functional Genomics Center Zurich (FGCZ), ETH Zurich / University of Zurich, Winterthurerstrasse 190, 8057 Zurich, Switzerland
[2] Swiss Institute of Bioinformatics (SIB), Quartier Sorge - Batiment Amphipole, 1015 Lausanne, Switzerland

[*] Correspondence: Witold E. Wolski <wew@fgcz.ethz.ch>

The *R* markdown file, with all the *R* code used to produce this pdf-document can be found here : prolfqua_supplement.Rmd. To replicated the analysis you need to install the *prolfquabenchmark* *R* package.

Material S1: How to Install *prolfqua* and *prolfquabenchmark*

To install the *prolfqua* package (ACS_JPR release), including all *R* package dependencies, execute in the *R* console:

```
remotes::install_github("https://github.com/fgcz/prolfqua/releases/tag/v1.0.0",
    dependencies = TRUE, build_vignettes=TRUE)
```

The R-markdown files with all the *R* code to run the benchmark for *prolfqua*, *msqrob2*, *MSStats* and *proDA* are available in the R package *prolfquabenchmark*.

```
remotes::install_gitlab("wolski/prolfquadata", host="gitlab.bfabric.org")
remotes::install_github("https://github.com/wolski/prolfquabenchmark/releases/tag/v0.2.0",
    dependencies = TRUE, build_vignettes=TRUE)
```

Material S2: Benchmark Vignettes (IonStar/MaxQuant)

Pre-build version of the vignettes, created to run the DEA benchmark, are available:

- Benchmarking of methods implemented in prolfqua using the IonStar dataset MaxQuant
- Benchmarking of MSstats using the IonStar dataset MaxQuant
- Benchmarking of proDA using the IonStar dataset MaxQuant
- Benchmarking of msqrob2 using the IonStar dataset MaxQuant

Material S3: DEA benchmark IonStar/MaxQuant/peptide.txt - Significance test

Table 1 summarizes *pAUC* of the DEA benchmark when using the IonStar/MaxQuant/peptide.txt data. Figure 5 in the Article, shows a barplot showing the $pAUC_{10}$.

We test if we should reject the null hypothesis that the area under the ROC curve at 0.1 FDR ($pAUC_{10}$), computed using the scaled p-values (see scaled.p.value's in Table 1), do not differ for *msqrob2*, *proDA*, and `prolfqua_merged`. To this task we use the function `roc.test`, that performs a bootstrap method test for a difference in the pAUC of the ROC curves, from the *R* package *pROC*. The Table 2 shows the p-values of the pairwise bootstrap test for partial areas under the ROC curves.

Material S4: DEA benchmark : CPTAC/MaxQuant/peptide.txt

We applied DEA using *msqrob2*, *proDA* and *prolfqua* ('prolfqua_merged') to the CPTAC/MaxQuant dataset and benchmarked the results. We are using the 'peptide.txt' file containing the peptide intensities.

The Rmarkdown file which generated these results, can be found here: "DEA benchmark IonStar/FragPipeV14/combined_protein.tsv"

Supplementary Figure 1 shows the partial area under the curve for *msqrob2*, *proDA* and *prolfqua*, when using the difference among the groups, the scaled p-values or the t-statistics to rank the proteins. For this dataset, the differences among groups show a higher performance than the t-statistic and scaled p-value. The *proDA* package performs slightly better then *prolfqua* or *msqrob* although the differences are between the $pAUC_{10}$ for the scaled p-value score are not statistically significant. Table 4 shows the results of the Bootstrap test for two ROC curves. We observe that for this benchmark data, we can not reject the null hypothesis that there are no significant differences among the $pAUC_{10}$ (ROC based on scaled p-value) for the three packages.

Supplementary Table 1: Results of the DEA beanchmark for IonStar/MaxQuant/peptide.txt

what	package	AUC	pAUC_10	pAUC_20
diff	msqrob2	92.7	66.1	78.6
diff	proDA	92.9	66.4	78.7
diff	prolfqua	91.9	64.8	77.3
diff	MSstats	92.6	64.7	77.9
scaled.p.value	msqrob2	93.0	73.1	79.6
scaled.p.value	proDA	93.0	72.6	79.1
scaled.p.value	prolfqua	92.5	72.3	78.8
scaled.p.value	MSstats	93.6	73.4	80.2
t_statistic	msqrob2	93.0	72.9	79.5
t_statistic	proDA	93.0	72.6	79.1
t_statistic	prolfqua	92.4	71.7	78.5
t_statistic	MSstats	93.6	73.4	80.3

Supplementary Table 2: p-values for pairwise comparsions of $pAUC_{10}$, for *msqrob2*, *proDA* and *prolfqua* for IonStar MaxQuant data.

pROC_test	p.value
msqrob2_vs_prolfqua	0.47
msqrob2_vs_proda	0.56
prolfqua_vs_proda	0.82

Supplementary Table 3: Results of the DEA beanchmark for CPTAC/MaxQuant/peptide.txt

what	package	AUC	pAUC_10	pAUC_20
diff	msqrob2	91.2	72.4	82.5
diff	proDA	89.9	79.4	84.8
diff	prolfqua	88.0	77.2	78.8
scaled.p.value	msqrob2	89.1	68.9	75.7
scaled.p.value	proDA	88.8	69.7	76.1
scaled.p.value	prolfqua	86.2	68.5	72.7
t_statistic	msqrob2	88.8	66.1	74.2
t_statistic	proDA	88.8	69.7	76.1
t_statistic	prolfqua	86.1	67.5	72.2

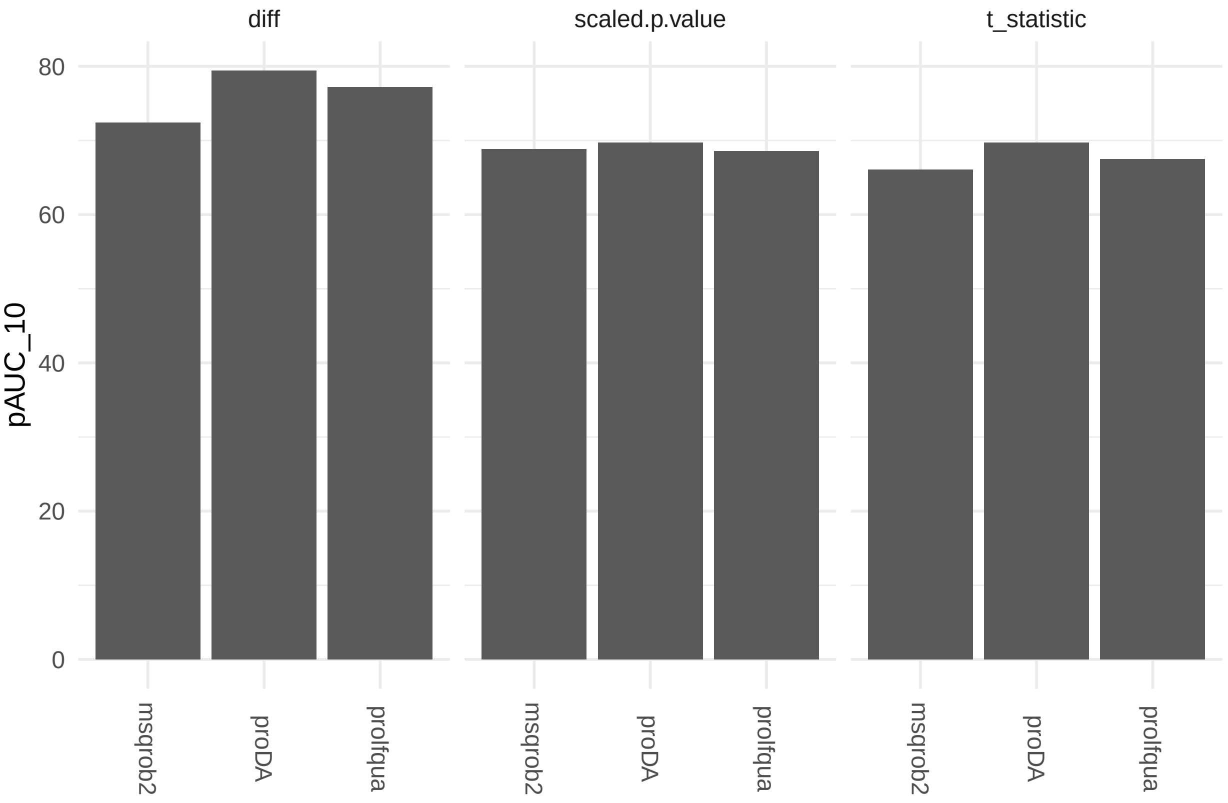

Supplementary Figure 1: Barplot showing the partial area under the ROC at 0.1 FDR for diff - differences among groups, scaled.p.value - $sign(diff) \cdot$ p-value and t-statistics. These results where obtained when using CPTAC/MaxQuant/peptide.txt as input.

Supplementary Table 4: p-values for pairwise comparsions of $pAUC_{10}$, for *msqrob2*, *proDA* and *prolfqua* CPTAC/MaxQuant/peptide.txt data.

pROC_test	p.value
msqrob2_vs_prolfqua	0.98
msqrob2_vs_proda	0.92
prolfqua_vs_proda	0.90

Supplementary Table 5: Results of the DEA beanchmark for IonStar/FragPipeV14/MSstats.tsv

what	package	AUC	pAUC_10	pAUC_20
diff	proDA	92.0	61.9	76.5
diff	prolfqua	93.7	69.0	79.4
scaled.p.value	proDA	94.2	80.0	83.6
scaled.p.value	prolfqua	95.1	81.6	85.2
t_statistic	proDA	94.2	80.0	83.6
t_statistic	prolfqua	94.8	78.7	83.8

Table 4 shows the results of the Bootstrap test for two ROC curves. We observe that for this benchmark data, we can not reject the null hypothesis that there are no significant differences among the $pAUC_{10}$ (ROC based on scaled p-value) for the three packages.

Material S5: DEA benchmark : IonStar/FragPipeV14/combined_protein.tsv

The Rmarkdown file which created these results, with more details about the IonStar FragPipe v14 dataset, and the data processing, can be found here: "DEA benchmark IonStar/FragPipeV14/combined_protein.tsv"

After filtering for two peptides per protein the dataset comprises of 3836 proteins. Since we are using the 'combined_protein.tsv' as input, comparison with neither *msqrob2* nor *MSstats* is possible. To fit the hurdle (*msqrob*) model, peptide intensities are required, while *MSstats* requires the 'MSstats.tsv' file, containing precursor level abundances. However, starting from protein level intensities has the advantage that the effect size estimates obtained from the model agree with protein abundances, a feature frequently requested by the biologist.

Supplementary Figure 2 shows the partial area under the curve for *proDA* and *prolfqua*, computed by ranking the proteins using the difference among groups (diff), the scaled p-value (scaled.p.value) and the t-statistics (t_statistics),

Table 6 shows the results of the Bootstrap test for the difference of two ROC curves. We observe that for this benchmark, there is no significant differences (at significance level of 0.1) among the $pAUC_{10}$, where the scaled p-value was use to rank the proteins, for the two packages.

Supplementary Table 6: p-values for pairwise comparsions of $pAUC_{10}$, for *proDA* and *prolfqua* CPTAC/MaxQuant data.

pROC_test	p.value
prolfqua_vs_proda	0.15

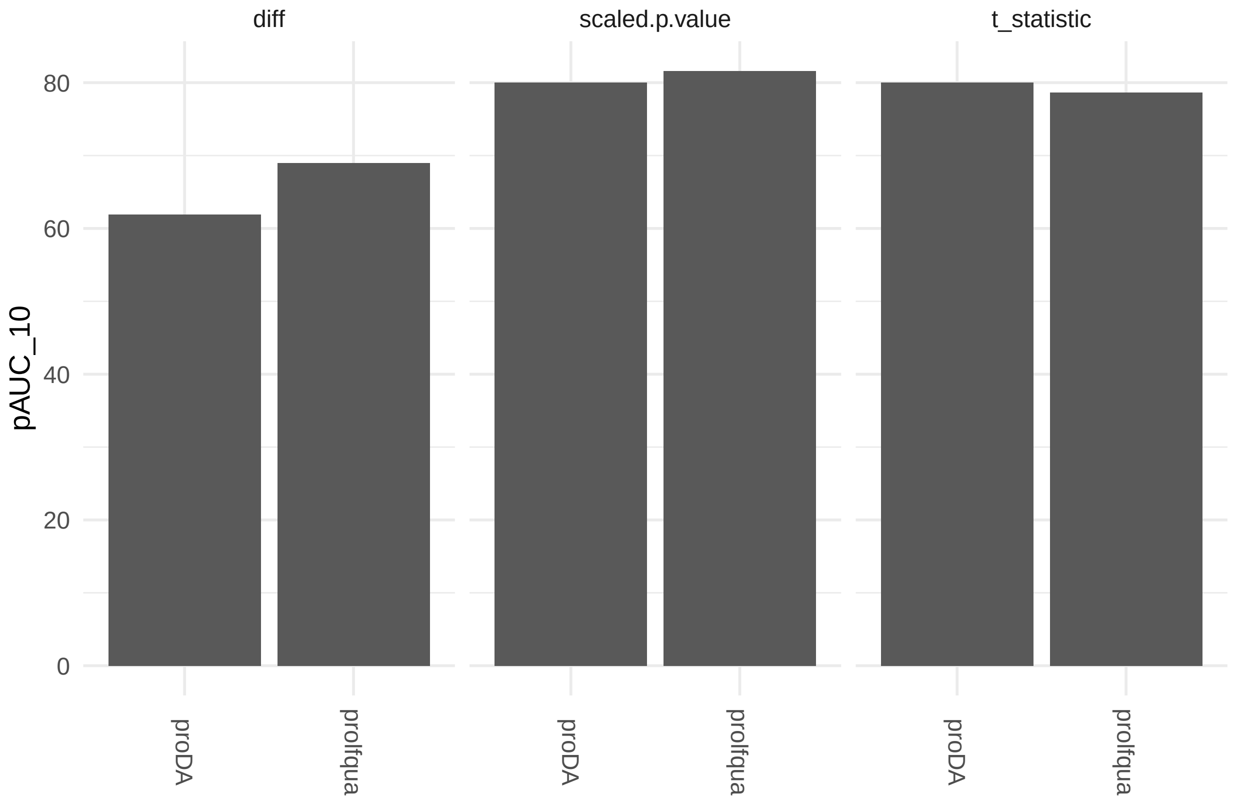

Supplementary Figure 2: Barplot showing the partial area under the ROC at 0.1 FDR ($pAUC_{10}$), for diff - difference among groups, scaled.p.value and t_statistic. These results where obtained when using IonStar/FragPipeV14/combined_protein.txt as input.

Material S6: DEA benchmark : IonStar/FragPipeV14/MSstats.tsv

The Rmarkdown file which created these results, with more details about the analysis, can be found here: "DEA benchmark IonStar/FragPipeV14/MSstats.tsv"

Since we are using the 'MSstats.tsv' file containing precursor level abundances as input, we can benchmark the DEA performed of *msqrob2*, *MSstats*, *proflqua*, and *proDA*. The dataset has 3899 proteins after filtering for two peptides per protein. Since we make four comparisons between groups, 15596 is the maximum possible number of effect size estimates. Supplementary Figure 3 shows the number of comparisons obtained. Supplementary Figure 2 shows the partial area under the curve for *proDA* and *prolfqua*, computed by ranking the proteins using the difference among groups (diff), the scaled p-value (scaled.p.value) and the t-statistics (t_statistics),

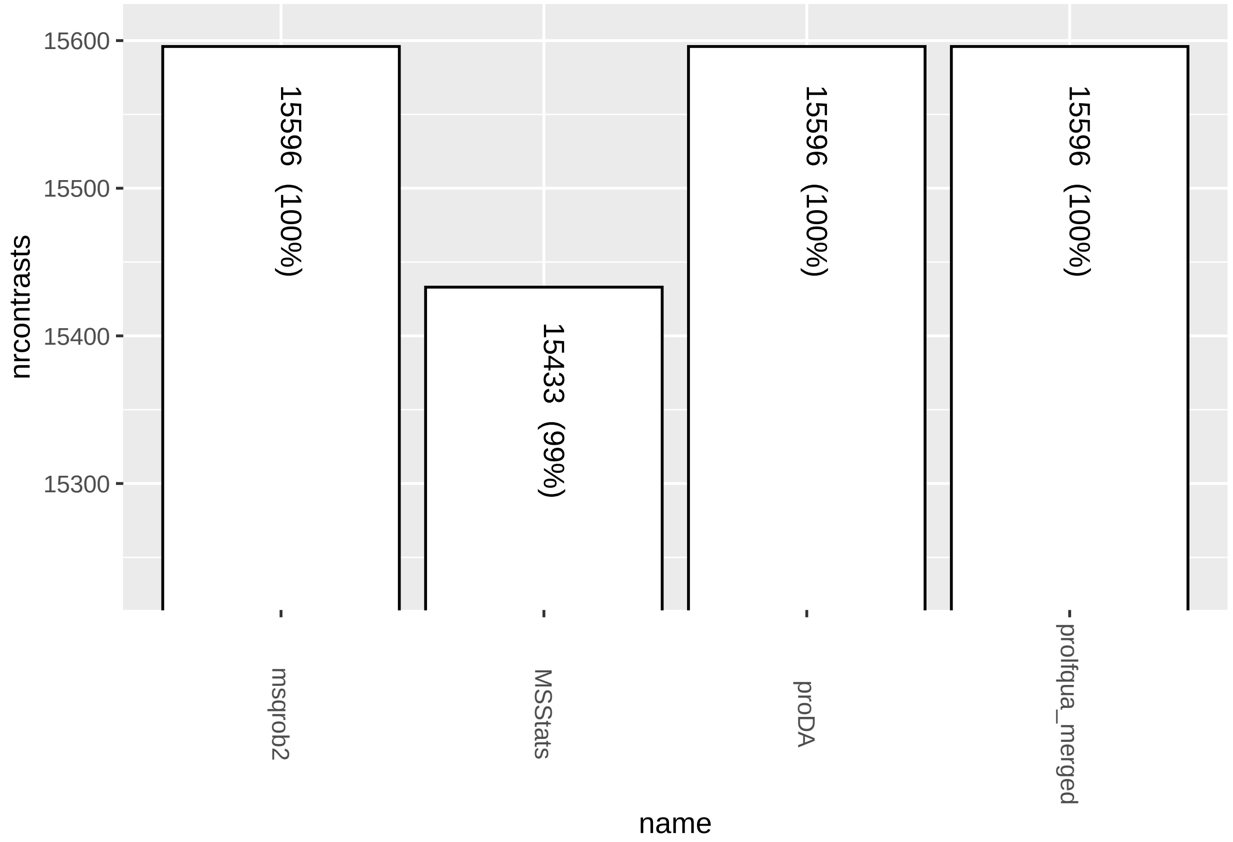

Supplementary Figure 3: Number of comparisons for each method for IonStar/FragPipeV14/MSstats.tsv data.

Material S7: Comparing DEA results for ***MaxQuant*** and ***FragPipe***

In the Article, we discussed other factors influencing the performance of the DEA analysis. One of these factors is the quantification software. Here we compare the benchmarking results obtained with MaxQuant and FragPipe. We see (Supplementary Figure 5) that the choice of the quantification software changes the partial area under the curve ($pAUC_{10}$) much stronger (by up to 8%) than the DEA analysis method (by about 4%). Furthermore, we observe that the DEA benchmark results differ depending on if 'MSstats.tsv' or 'combined_protein.tsv' is used as input (see Figure 5 Left Panel, methods *proDA* and *prolfqua*). Supplementary Table 8 shows the p-values when we test if the $pAUC_{10}$, summarizing the DEA benchmark results, significantly differ between the *MaxQuant* and *FragPipe*. At significance level of 0.01 we will reject the null hypothesis that there is no difference between *FragPipe* and *MaxQuant*.

Supplementary Table 7: Results of the DEA beanchmark for IonStar/FragPipeV14/MSstats.tsv

what	package	AUC	pAUC_10	pAUC_20
diff	proDA	94.9	75.9	85.4
diff	prolfqua	95.1	75.3	85.0
diff	msqrob2	95.2	77.0	86.1
diff	MSstats	95.4	72.9	84.4
scaled.p.value	proDA	95.1	80.0	84.6
scaled.p.value	prolfqua	96.1	83.4	87.9
scaled.p.value	msqrob2	96.1	83.4	87.9
scaled.p.value	MSstats	96.6	81.5	87.4
t_statistic	proDA	95.1	80.0	84.6
t_statistic	prolfqua	96.1	83.1	87.8
t_statistic	msqrob2	96.1	83.2	87.8
t_statistic	MSstats	96.6	81.5	87.4

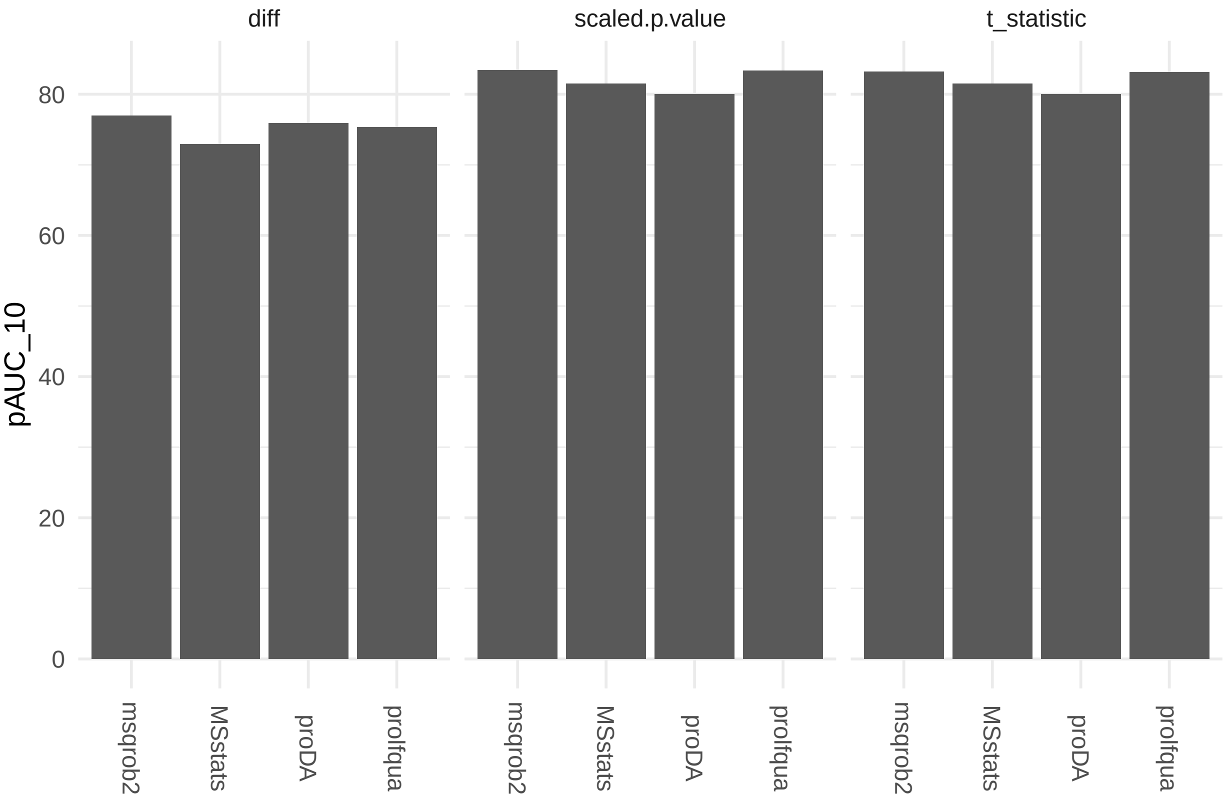

Supplementary Figure 4: Barplot showing the partial area under the ROC at 0.1 FDR ($pAUC_{10}$), for diff - difference among groups, scaled.p.value and t_statistic. These results where obtained when using IonStar/FragPipeV14/MSstats.tsv as input.

Supplementary Table 8: p-values for pairwise comparsions of $pAUC_{10}$ for MaxQuant and FragPipe based DEA analysis with prolfqua. We compared the pAUC for the difference estimates (diff) and for the scaled.p.values.

pROC_test	p.value
MQ_vs_FP_scaled.p.value	0
MQ_vs_FP_diff	0

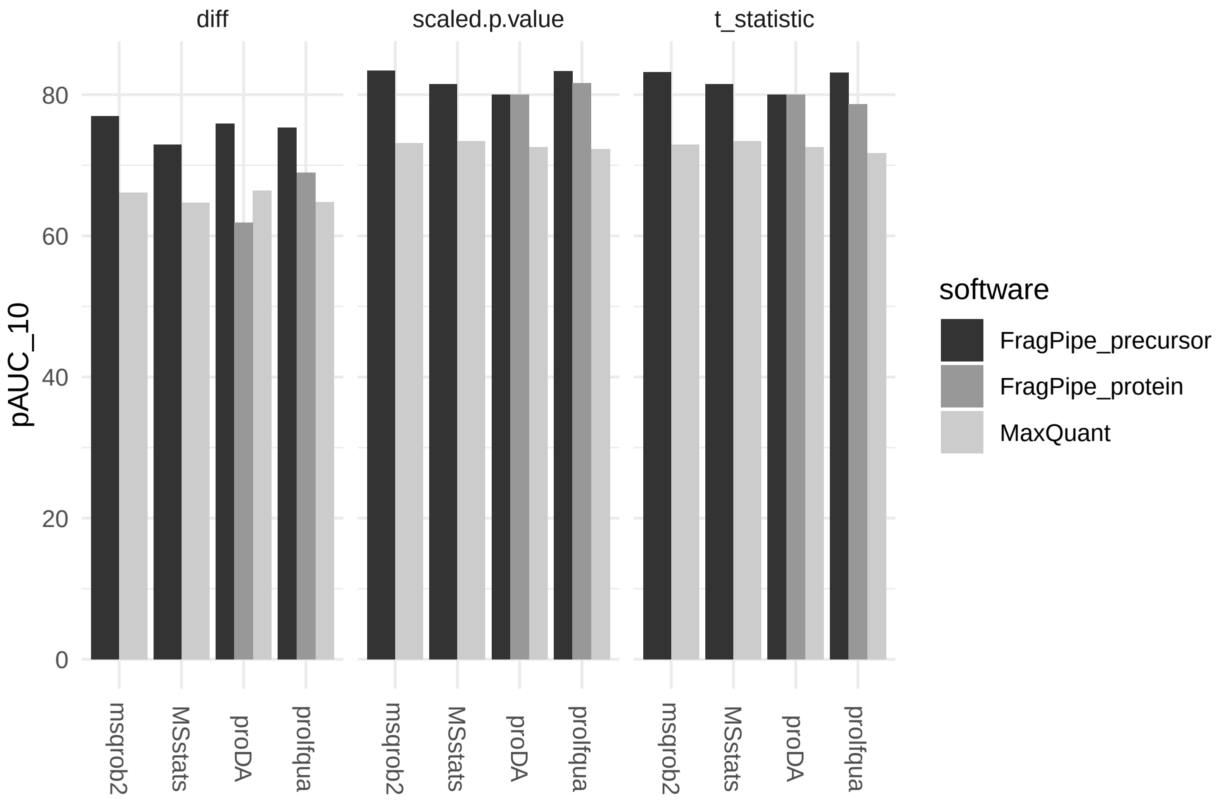

Supplementary Figure 5: Barplot, showing the DEA results ($pAUC_{10}$ - y axis) for four DEA methods and two quantification methods (FragPipeV14/MSstats.tsv in black, FragPipeV14/combined_protein.txt dark gray and MaxQuant/peptide.txt in gray).

Material S8: Estimating A_{LOD}

The main text describes how we estimate the abundance at the detection limit A_{LOD}. Supplementary Figure 6 shows the distribution of the average abundance within a group for all proteins. The red density shows the distribution for those proteins without a missing value, and the olive, turquoise, and lilac density show for proteins with 1,2 and 3 missing values, respectively. We see that the proteins with three missing values have, on average lower abundances than those with no missing values. We are using the median of the proteins with a single observation per group; the lila density, as the A_{LOD} estimate.

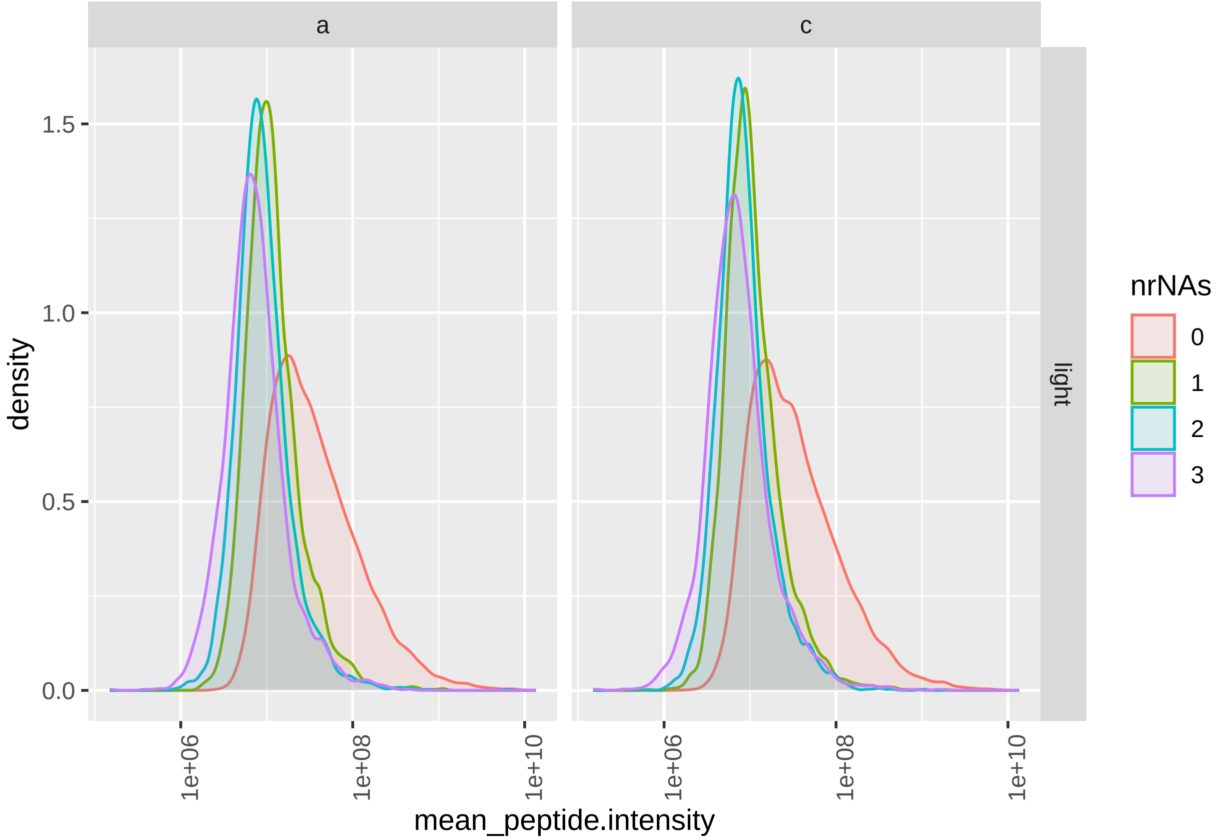

Supplementary Figure 6: Distribution of the average abundance within group a and c for all proteins. Each density are color coded depending the number of missing observations per protein in a group.

Material S9: The probabilities produced by ROPECA are not p-values

In the main text discuss that the properties of the Beta distribution-based probabilities, computed from peptide level p-values, using the ROPECA method are not well understood. We are running here a simulation experiment, which shows that if we start with a dataset where the null hypothesis is valid for all the peptides, i.e., the p-values are uniformly distributed, the produced Beta distribution-based probabilities are not uniformely distributed.

For all the peptides (`nrPep = 10000`) $H0$ is true. We know that if $H0$ is true, the distribution of the $p-values$ will be uniform. (Supplementary Figure 7 left Panel). Therefore, we can omit the step of generating the data from $H0$ and computing the p-values but simulate the p-values directly by sampling from the uniform distribution (`runif(nrPep)`).

By sampling 800 protein id's 10000 times (with replacement), we assign the peptides to proteins ('sample(1:800,

size = nrPep, replace = TRUE, . . .)'). To make the data more realistic, that is that some proteins have a lot of peptides, and many have just a few peptides; the density of sampling the protein Id is exponential (`dexp(seq(0,5,length = 800))`). Supplementary Figure 7 center panel shows the number of proteins as a function of the number of peptides per protein. Afterward, we apply the ROPECA methods as discussed in the methods section of the manuscript. Supplementary Figure 7 shows the distribution of the Beta distribution-based probabilities, which is not uniform. Hence, they do not have the same interpretation as p-values, i.e., the probability of falsely rejecting the $H0$ if $H0$ is true.

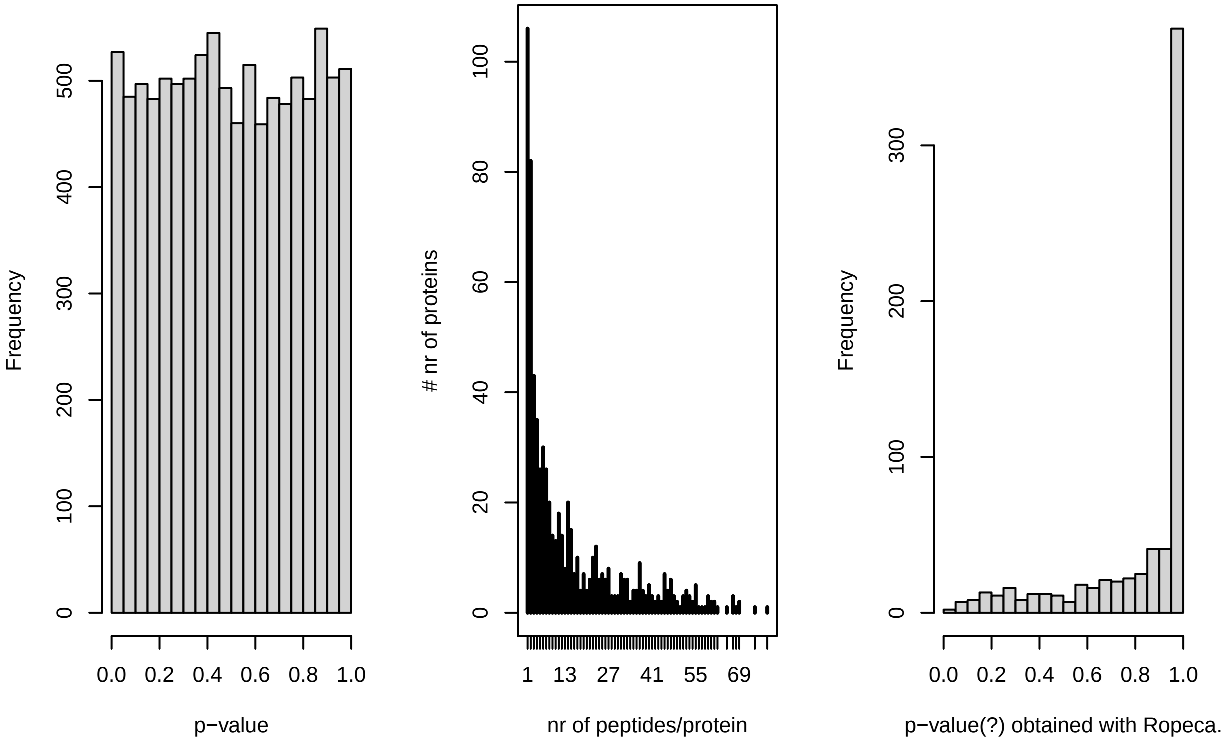

Supplementary Figure 7: Left panel - distibution of peptide level p.values. Center panel - number of proteins as a function of the number of peptides per protein. Right panel - distribution of protein level p-values(?) obtained with the ropeca method.

Material S10: Specifying Contrasts for Models with two Factors and Interaction Term

The following code illustrates how to specify a model with two factors and an interaction term and define the differences we want to estimate. The example dataset was acquired in two batches (p2370, p2691). In each, we measured Yeast samples grown under two different conditions (Glucose and Ethanol).

To estimate treatment differences, we first specify the linear model that explains the observed protein abundances (transformedIntensities) using the explanatory variables `condition_` and `batch_` and the interaction term `condition_:batch_`. Then, after fitting the model, we can estimate the treatment difference among the groups `Ethanol` and `Glucose` defined by the factor `condition` and similarly among the groups defined by `batch_`. Furthermore, we can examine differences between `Ethanol` and `Glucose` within each batch. We also can assess if these differences are the same or are different in both batches, i.e., if there is an interaction between condition and batch. We also show the code to compute the array of weights c used to multiply the model coefficient β.

Supplementary Table 9: Weights c for each of the contrasts (rows) which will be applied to the model parameter (columns).

	(Intercept)	condition_Glucose	batch_p2691	condition_Glucose:batch_p2691
diff_condition	0	-1	0	-0.5
diff_batch	0	0	-1	-0.5
diff_within_p2370	0	1	-1	0.0
diff_within_p2691	0	1	0	1.0
diff_of_difference	0	0	-1	-1.0

```
Yeast2Factor <- prolfqua::prolfqua_data("data_Yeast2Factor")
Yeast2Factor$data |> dplyr::select(condition_, batch_) |> dplyr::distinct()
```

```
## # A tibble: 4 x 2
##   condition_ batch_
##   <chr>      <chr>
## 1 Glucose    p2370
## 2 Ethanol    p2370
## 3 Ethanol    p2691
## 4 Glucose    p2691
```

```
mlm <- prolfqua::strategy_lm(
  transformedIntensity ~ condition_ + batch_ + condition_:batch_
  )
mm <- prolfqua::build_model(Yeast2Factor$data, mlm, subject_Id = "protein_Id")

Contrasts <- c(
"diff_condition" = "condition_Ethanol - condition_Glucose",
"diff_batch" = "batch_p2370 - batch_p2691",
"diff_within_p2370" =
  "`condition_Glucose:batch_p2370` - `condition_Ethanol:batch_p2691`",
"diff_within_p2691" =
  "`condition_Glucose:batch_p2691` - `condition_Ethanol:batch_p2691`",
"diff_of_difference" = "diff_within_p2370 - diff_within_p2691")

linfct <- prolfqua::linfct_from_model(mm$modelDF$linear_model[[1]],
                                      as_list = FALSE)
mC <- prolfqua::linfct_matrix_contrasts(linfct, Contrasts)
## see Table 3 for content of mC
```

Table 9 shows weights c for each of the five contrasts we specified.

Given the weights c contrasts from model parameters obtained with *proDA*, or *msqrob2* can be estimated, which will enables us to implement adapters to *proDA* or *msqrob2*.

For more details see *prolfqua* vignette: "Modelling dataset with two Factors".

Material S11: Creating a prolfqua configuration

The following code demonstrates how we use *prolfqua* to analyze protein intensities reported in the *FragPipe* 'combined_protein.tsv' file. First, we create a tidy table containing the protein abundances by reading the `combined_protein.tsv` file using `tidy_FragPipe_combined_protein`. Then, we read the sample annotation from the file `annotation.xlsx` file. Next, we create an `AnalysisTableAnnotation` R6 object. Bottom-up proteomics data is hierarchical, i.e., a protein has peptides, peptides might be modified, etc. Therefore, the

AnalysisTableAnnotation has a hierarchy field storing a list with an entry for each hierarchy level. Since combined_portein.tsv only holds protein level data, the hierarchy list has one element, and we use it to specify which column contains the protein identifiers. We also need to define which column contains the protein abundances we want to use for the data analysis. Finally, we have to specify which columns contain the explanatory variables of the analysis. The AnalysisTableAnnotation has the field factors, a list with as many entries as explanatory variables. Here we include two explanatory variables, the dilution, specified in the column 'sample', and 'run' stored in the column 'run__ID', representing the order of the measurement.

```
datadir <- file.path(find.package("prolfquadata") , "quantdata")
inputFragfile <-  file.path(datadir, "MSFragger_IonStar2018_PXD003881.zip")
inputAnnotation <- file.path(datadir, "annotation_Ionstar2018_PXD003881.xlsx")
# read input annotation
annotation <- readxl::read_xlsx(inputAnnotation)

protein <- tibble::as_tibble(
    read.csv(unz(inputFragfile,"IonstarWithMSFragger/combined_protein.tsv"),
             header = TRUE, sep = "\t", stringsAsFactors = FALSE))

# read combined_protein.tsv
protein <- prolfqua::tidy_FragPipe_combined_protein_deprec(protein)
# remove proteins identified by a single peptide
protein <- protein |>
  dplyr::filter(unique.stripped.peptides > 1)

# annotate the data
merged <- dplyr::inner_join(annotation, protein)
atable <- prolfqua::AnalysisTableAnnotation$new()
atable$fileName = "raw.file"
# specify column containing protein identifiers
atable$hierarchy[["protein_Id"]] = "protein"

# column with protein abundances
atable$set_response("total.intensity")

# the factors of the analysis
atable$factors[["dilution."]] = "sample"
atable$factors[["run"]] = "run_ID"

config <- prolfqua::AnalysisConfiguration$new(atable)

adata <- prolfqua::setup_analysis(merged, config)
lfqdata <- prolfqua::LFQData$new(adata, config)
# show number of proteins in the dataset
lfqdata$hierarchy_counts()
```

```
## # A tibble: 1 x 2
##   isotopeLabel protein_Id
##   <chr>             <int>
## 1 light              4461
```

Miscellaneous

```
file.path("graphics/hexstrickerPRL2.png") |>
  knitr::include_graphics()
```

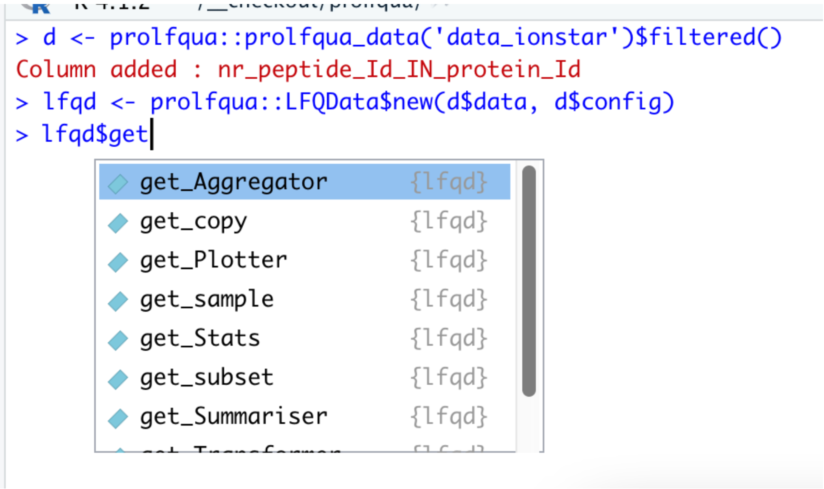

Supplementary Figure 8: The screenshot displays the command-line completion (tab completion) of RStudio on the `prolfqua::LFQData` R6 object. In the example, it shows the getter methods of the object.

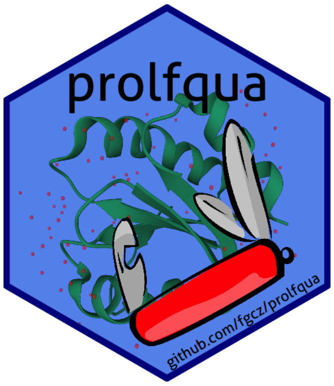

Supplementary Figure 9: Sticker maintainer: Witold E. Wolski; License: Creative Commons Attribution CC-BY. Feel free to share and adapt, but don't forget to credit the author.

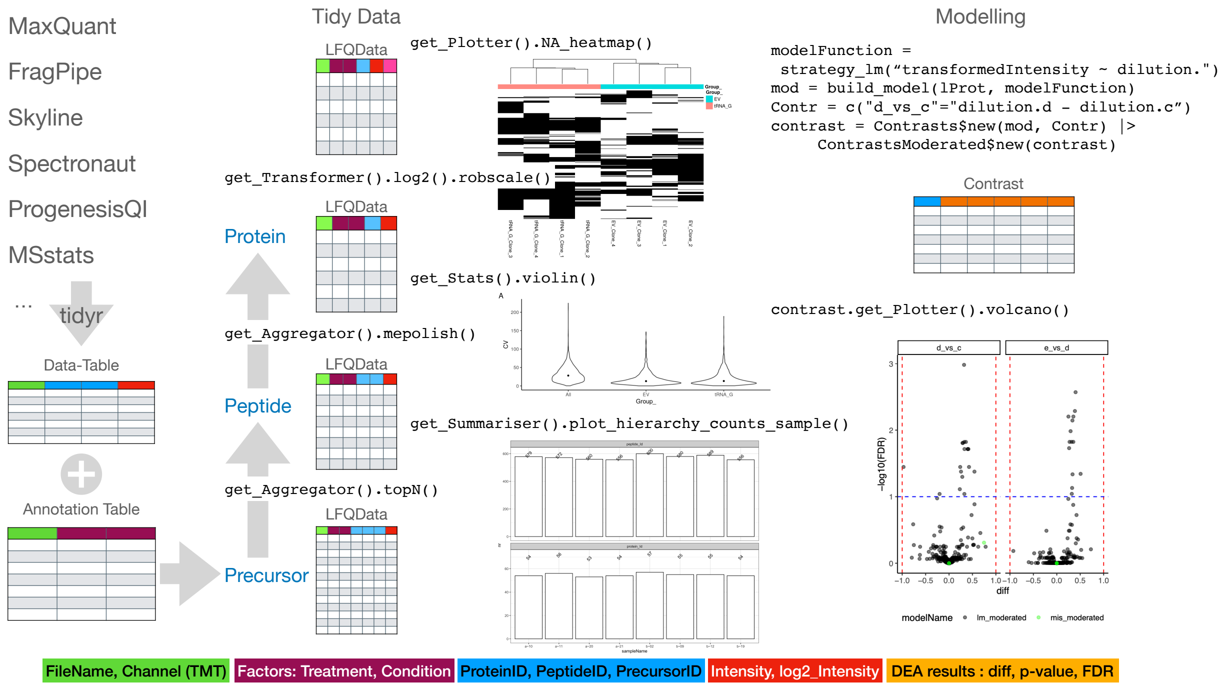

Supplementary Figure 10: The figure illustrates some possible data analysis procedures that can be achieved with only very few functions from prolfqua. As input different levels from a variety of quantitation tools are accepted and along with an annotation table configured for prolfqua using either pre-built import functions or made ready with a little reshaping. In prolfqua (depending on the input), one can choose roll-up procedures and perform basic steps of mass spectrometry-based data processing steps such as quality control, data normalization, protein aggregation, as well as statistical modeling, hypothesis testing, and sample size estimation. At all levels, one can always use functions to show diagnostic plots or even inspect individual proteins. Furthermore, prolfqua supports multiple models for differential expression analysis such as linear (mixed effect) models and allows to specify multiple contrasts.

Session info

R version 4.2.1 (2022-06-23)

Platform: aarch64-apple-darwin20 (64-bit)

locale: en_US.UTF-8||en_US.UTF-8||en_US.UTF-8||C||en_US.UTF-8||en_US.UTF-8

attached base packages: *stats*, *graphics*, *grDevices*, *utils*, *datasets*, *methods* and *base*

loaded via a namespace (and not attached): *httr(v.1.4.5)*, *tidyr(v.1.3.0)*, *jsonlite(v.1.8.4)*, *viridisLite(v.0.4.1)*, *carData(v.3.0-5)*, *conflicted(v.1.2.0)*, *BiocManager(v.1.30.19)*, *highr(v.0.10)*, *pander(v.0.6.5)*, *cellranger(v.1.1.0)*, *yaml(v.2.3.7)*, *progress(v.1.2.2)*, *ggrepel(v.0.9.3)*, *pillar(v.1.8.1)*, *backports(v.1.4.1)*, *glue(v.1.6.2)*, *pROC(v.1.18.0)*, *digest(v.0.6.31)*, *RColorBrewer(v.1.1-3)*, *ggsignif(v.0.6.4)*, *rvest(v.1.0.3)*, *colorspace(v.2.1-0)*, *htmltools(v.0.5.4)*, *plyr(v.1.8.7)*, *pkgconfig(v.2.0.3)*, *pheatmap(v.1.0.12)*, *broom(v.1.0.3)*, *bookdown(v.0.32)*, *purrr(v.1.0.1)*, *scales(v.1.2.1)*, *webshot(v.0.5.4)*, *svglite(v.2.1.0)*, *tibble(v.3.1.8)*, *generics(v.0.1.3)*, *farver(v.2.1.1)*, *car(v.3.1-1)*, *ggplot2(v.3.4.1)*, *ellipsis(v.0.3.2)*, *ggpubr(v.0.6.0)*, *cachem(v.1.0.7)*, *withr(v.2.5.0)*, *lazyeval(v.0.2.2)*, *cli(v.3.6.0)*, *magrittr(v.2.0.3)*, *crayon(v.1.5.2)*, *readxl(v.1.4.2)*, *memoise(v.2.0.1)*, *evaluate(v.0.20)*, *fansi(v.1.0.4)*, *MASS(v.7.3-58.1)*, *rstatix(v.0.7.2)*, *forcats(v.1.0.0)*, *xml2(v.1.3.3)*, *prettyunits(v.1.1.1)*, *tools(v.4.2.1)*, *data.table(v.1.14.8)*, *hms(v.1.1.2)*, *BiocStyle(v.2.24.0)*, *lifecycle(v.1.0.3)*, *stringr(v.1.5.0)*, *plotly(v.4.10.1)*, *munsell(v.0.5.0)*, *kableExtra(v.1.3.4)*, *compiler(v.4.2.1)*, *systemfonts(v.1.0.4)*, *prolfqua(v.1.1.1)*, *rlang(v.1.0.6)*, *grid(v.4.2.1)*, *rstudioapi(v.0.14)*, *htmlwidgets(v.1.6.1)*, *labeling(v.0.4.2)*, *rmarkdown(v.2.20)*, *gtable(v.0.3.1)*, *abind(v.1.4-5)*, *R6(v.2.5.1)*, *gridExtra(v.2.3)*, *knitr(v.1.42)*, *dplyr(v.1.1.0)*, *fastmap(v.1.1.1)*, *utf8(v.1.2.3)*, *stringi(v.1.7.12)*, *Rcpp(v.1.0.10)*, *png(v.0.1-7)*, *vctrs(v.0.5.2)*, *tidyselect(v.1.2.0)* and *xfun(v.0.37)*
